# Maternal inflammatory markers for chorioamnionitis in preterm prelabour rupture of membranes: a systematic review and meta-analysis of diagnostic test accuracy studies

**DOI:** 10.1186/s13643-020-01389-4

**Published:** 2020-06-12

**Authors:** Angela Koech Etyang, Geoffrey Omuse, Abraham Mwaniki Mukaindo, Marleen Temmerman

**Affiliations:** 1 Department of Obstetrics and Gynaecology, Aga Khan University, P.O. Box 30270-00100, Nairobi, Kenya; 2 Department of Pathology, Aga Khan University, P.O. Box 30270-00100, Nairobi, Kenya

**Keywords:** Inflammatory markers, Chorioamnionitis, C-reactive protein, Procalcitonin, Interleukin 6

## Abstract

**Background:**

There is no consensus on the role of inflammatory markers in identifying chorioamnionitis in preterm prelabour rupture of membranes (PPROM). We set out to evaluate the accuracy of maternal blood C-reactive protein (CRP), procalcitonin and interleukin 6 (IL6) in diagnosis of histological chorioamnionitis and/or funisitis (HCA/Funisitis) in PPROM.

**Methods:**

We searched MEDLINE, EMBASE and The Cochrane Library from inception to January 2020 for studies where maternal blood CRP, procalcitonin or IL6 was assessed against a reference standard of HCA/Funisitis in PPROM. The Quality Assessment of Diagnostic Accuracy Studies 2 (QUADAS-2) tool was used to assess methodological quality. Hierarchical summary receiver operating characteristic (SROC) models were used to construct summary curves. Bivariate models were used to obtain summary estimates for studies with the same cut-off.

**Results:**

We included 23 studies reporting HCA/Funisitis in 902 of 1717 women, median prevalence 50% (inter-quartile range 38–57). Of these studies, 20 were prospective cohort design and 3 were retrospective cohort. Eleven studies reported the index test against a reference standard of HCA and/or funisitis, 10 reported HCA alone and 2 reported funisitis alone. Many studies had high risk of bias scores on the QUADAS-2 assessment but low concerns for applicability. Sensitivity and specificity for CRP ≥ 20 mg/L (5 studies, 252 participants) was 59% (95% CI 48–69) and 83% (95% CI 74–89) respectively. SROC curves are provided for each index test. At selected specificity of 80%, the sensitivities for CRP (all cut-offs, 17 studies, 1404 participants), PCT ( all cut-offs, 6 studies, 231 participants) and IL6 (all cut-offs, 5 studies, 299 participants) were 59%(95% CI 52–68), 56%(95% CI 50–69) and 52% (95% CI 50–86) respectively.

**Conclusions:**

There is insufficient evidence to support use of CRP, procalcitonin or IL6 in maternal blood for diagnosis of HCA/Funisitis in PPROM. This review followed recommended methodology and data analytic methods that made the most of the data regardless of the different cut-offs used. However, the evidence is based on few studies with generally small sample sizes, poor-quality scores and substantial heterogeneity. There is a need for good-quality diagnostic accuracy studies to better assess the role of these biomarkers in PPROM.

**Systematic review registration:**

PROSPERO registration number: CRD42015023899, registered on 8 October 2015.

## Background

In preterm prelabour rupture of membranes (PPROM), the decision for delivery is a delicate balance that considers risks of preterm birth versus risks of infection from continuing pregnancy [1, 2]. Typically, expectant management is carried out until the patient develops clinical signs suggestive of infection or until an appropriate gestation for safe delivery is reached. If clinical features of infection or inflammation are detected, then usually delivery is initiated. These clinical features can be thought of as an existing test. Inflammatory markers may form a suitable replacement test in place of the clinical features as the latter often become evident late or remain absent even in the presence of chorioamnionitis [3]. If inflammatory markers assayed in maternal blood are found to be sufficiently accurate in the diagnosis of chorioamnionitis, they can influence clinical decision-making and reduce reliance on clinical features alone. Early diagnosis of infection can advise therapeutic interventions such as delivery and antibiotic administration [4].

Maternal serum offers a readily accessible biological sample for assay of inflammatory markers and is preferred over alternative samples such as amniotic fluid which are harder to obtain [5] and not always available in non-specialist centres. Cord blood is an alternative sample, but its availability only after delivery precludes its use in decision-making during pregnancy.

There is no consensus on a suitable reference standard for diagnosis of chorioamnionitis [5–8]. We opted to use histologic chorioamnionitis (HCA) and/or funisitis as the reference standard for this review because standard criteria for ascertainment have existed for many years [9], its assessment is objective where these criteria are applied and there is good correlation with neonatal outcomes [10].

Several studies have evaluated maternal inflammatory markers for diagnosis of chorioamnionitis in PPROM with varying results and recommendations. Current guidelines [1, 4] do not recommend use of these markers alone for diagnosing infection in PPROM, but despite this, many clinicians continue to use these tests in PPROM with the results potentially influencing clinical decision-making [11]. Older reviews suggested CRP is useful in diagnosis of chorioamnionitis [12], but more recent systematic reviews [6–8] give no clear evidence for this recommendation. Prior systematic reviews have evaluated the role of C-reactive protein (CRP) in PPROM [6, 7] and do not recommend its use for predicting chorioamnionitis. However, these reviews were based on few studies [6–8], demonstrated marked heterogeneity [6, 7] and used data analysis methods that are not recommended [6, 8]. Several studies assessing CRP and other inflammatory markers have since been published.

The objective of this review was to evaluate the accuracy of maternal blood inflammatory markers: C-reactive protein (CRP), procalcitonin (PCT) and interleukin 6 (IL6) in the diagnosis of histologic chorioamnionitis and/or funisitis in PPROM and to assess the sources of heterogeneity in estimates of diagnostic accuracy.

## Methods

This systematic review of diagnostic accuracy employed methodological approaches recommended in the Cochrane Handbook for Systematic Reviews of Diagnostic Test Accuracy [13] and followed a prospectively prepared protocol [14] registered with PROSPERO CRD42015023899. This report complies with the Preferred Reporting Items for Systematic Reviews and Meta-analyses of Diagnostic Test Accuracy Studies, the PRISMA-DTA statement [15], and PRISMA-DTA checklists are provided as Additional file 1.

The inclusion criteria were studies of pregnant women with PPROM before 37 completed weeks of gestation. The tests of interest were CRP, PCT and IL6 performed on a maternal blood sample obtained prior to delivery, with any cut-off and any method of assay. The reference standard for chorioamnionitis was histologic chorioamnionitis and/or funisitis (HCA/Funisitis)—where a definition or diagnostic criteria was provided or a specification of histologic or microscopic assessment of the placenta was indicated or where the placenta was assessed by a pathologist. Any study design where the results of the index test were compared with the reference standard and reported data allowed extraction of 2 × 2 data was eligible.

We aimed to identify relevant studies published in peer-reviewed journals. We searched MEDLINE, EMBASE and The Cochrane Library from inception to 5 Jan 2020 and performed manual searches on reference lists of included articles and previous related reviews. The search strategy included a combination of subject headings and free-text terms related to the index test and target population only. We did not use any filters or search terms for the study design [16, 17] nor did we include the term ‘diagnostic study’. There were no restrictions for language, publication dates or geographical setting in the electronic search. Where the database allowed, the limit for ‘Humans’ was applied. The search strategy is provided in Additional file 2.

Initial screening of titles and/or abstracts and subsequent in-depth review of full texts were done independently by 2 reviewers each (AKE, GO and AMM). Disagreements were resolved by consensus that included a third reviewer (AMM). Despite no restrictions for language in the electronic search and abstract screening, studies with non-English/non-French full texts were excluded due to anticipated difficulties in obtaining translations. Data extraction was done independently by 2 reviewers (AKE and GO) using a custom data extraction form that was piloted on 3 randomly selected eligible studies. Extracted fields included study characteristics (study design, setting, year of study, inclusion criteria, gestational age range), characteristics of the index tests (index test, method of assay, cut-off(s), timing of index test relative to delivery), clinical management of participants (antibiotic use, steroids, tocolysis) and indices of diagnostic accuracy. True positive, true negative, false positive and false negative values (2 × 2 data) for each test in each study and for each cut-off reported were extracted or calculated from indices of diagnostic accuracy provided. In studies with a wide range of clinical diagnoses (e.g. including preterm labour with intact membranes) or wide gestational age range (e.g. including term PROM), 2 × 2 data was extracted for the PPROM subgroup where this was reported separately. Authors of otherwise eligible studies but with missing, unclear or conflicting 2 × 2 data were contacted by email.

A review-specific checklist derived from the Quality Assessment of Diagnostic Accuracy Studies 2 (QUADAS-2) [18] tool was used to assess the methodological quality of included studies. Assessments were done by 2 reviewers (GO and AKE) independently with disagreements resolved by consensus. Studies with a low risk of bias in patient selection were those that employed consecutive or random sampling and excluded women with clinical features of chorioamnionitis and/or preterm labour at the time of presentation with PPROM. Patient selection criteria that were potential sources of bias included selecting patients based on availability of other tests or completeness of records, restricting patients to a particular duration of PPROM and excluding women with common pregnancy-related or medical conditions. For the reference standard, objective and blinded assessment of the placenta was considered to have low risk of bias. The study flow and timing was considered to be of low risk of bias if the interval between blood sampling and delivery (proxy for placental assessment) was ≤ 72 h and if data were analysed and reported for ≥ 90% of included participants.

We obtained study estimates of sensitivity, specificity and corresponding 95% confidence intervals (CI) and displayed these on coupled forest plots. Meta-analysis was carried out if the number of studies in each index test category was ≥ 3. All reference standards were considered together as one. Summary receiver operator characteristic (SROC) curves were constructed for each test regardless of cut-off using the Rutter and Gatsonis’ Hierarchical SROC (HSROC) model [19]. HSROC analysis was conducted using the NLMIXED procedure in SAS® (University Edition 2016, Cary, NC), and the parameter estimates obtained were then inputted into Cochrane Review Manager (RevMan, version 5.3, Copenhagen) for construction of the curves [20]. The HSROC analysis is a random effects model, and it accounts for the correlation between sensitivity and specificity across the studies with changes in threshold [19, 20]. It makes the most use of the data as studies are pooled regardless of differences in cut-offs [20]. For studies using the same cut-off, we used bivariate analysis to obtain summary sensitivity and specificity and corresponding 95% CIs. To aid understanding of the findings, we derived normalised frequencies assuming a patient population of 100 women and a prevalence obtained from the median prevalence of the included studies [21]. For SROC curves, we chose a false positive rate and derived corresponding sensitivity and confidence intervals from the model [21].

Heterogeneity assessment for studies using the same cut-off was carried out by visually inspecting the 95% prediction regions on SROC curves [20]. For the other studies, further exploration for causes of heterogeneity was carried out where the number of studies exceeded 5 and each subgroup had at least 2 studies. We aimed to evaluate the following as possible sources: assay type, pre-specified cut-off, interval between sampling and delivery and the risk of bias score in the patient selection domain of the QUADAS-2. These characteristics were added as binary covariates to the HSROC models in SAS® (University Edition 2016, Cary, NC). Pairs of SROC curves were constructed by inputting the parameters into Cochrane Review Manager (RevMan, version 5.3, Copenhagen). For simplicity, the shape parameter was assumed to be the same in the 2 subgroups. Chi-squared test was used to compare the 2-Log likelihoods to test for differences in SROC curves between subgroups. Covariates were applied to the model one at time and curves compared for each characteristic in turn. We did not construct models with more than one covariate due to limited power in the setting of few studies [20, 22].

We performed sensitivity analysis to investigate the possible influence of including studies with a narrower gestational age range (limiting the review to studies with gestational age above 24 weeks), year of publication (limiting the review to studies published after year 2000) and limiting the review to studies with low concerns for applicability on the patient selection domain of the QUADAS-2 assessment. Pairs of SROC plots were constructed and comparison done visually [20]. No assessment of publication bias was performed as included studies were few or too heterogeneous [23, 24].

## Results

### Results of the search

**Fig. 1:**
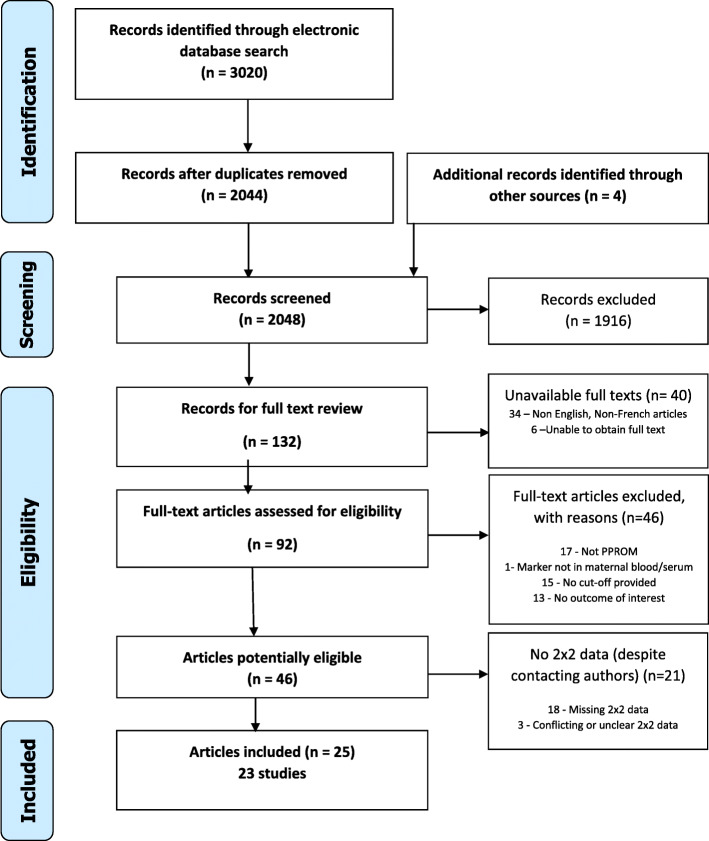
Study flow diagram. PPROM, preterm prelabour rupture of membranes. HCA, histologic chorioamnionitis. Figure modified from the PRISMA statement [25]

The search yielded 3020 unique records of which 25 (25 publications, 23 unique studies) were included (Fig. 1). Twenty-one of the 46 potentially eligible studies were excluded due to missing or unclear 2 × 2 data. More information on these studies is provided in Additional file 3. No additional data was obtained from contacted authors.

### Characteristics of included studies

The studies were published between 1983 and 2019 and conducted in 13 countries. Twenty studies were prospective cohort design and 3 studies retrospective cohort design. All were conducted in hospital inpatient settings with majority at teaching/university hospitals. In total, there were 1717 participants, 902 of whom had HCA/funisitis; median prevalence 50%; and inter-quartile range 38% to 57%. Characteristics of included studies are summarised in Table 1.

**Table 1:** Characteristics of included studies

Study (reference)	Country	Study design	No. of participants (excluded)*	Diagnosis of PPROM; confirmation of PPROM	GA range in inclusion criteria (weeks)	Clinical management of PPROM			Index test(s)	Reference standard	Number with outcome/total (prevalence %)
						Antibiotics	Steroids	Tocolytics			
Farb et al. 1983 [26]	USA	Prospective cohort	31 (7)	Examination or nitrazine positive or fern test positive	20 to 36	NR	Yes	Yes	CRP	HCA and funisitis	5/24 (21)
Hawrylyshyn et al. 1983 [27]	Canada	Prospective cohort	54 (2)	Nitrazine positive or pooling of AF	20 to 34	None	Yes	Selective	CRP	HCA	26/52 (50)
Ismail et al. 1985 [28]	USA	Prospective cohort	100 (0)	Pooling of AF or nitrazine positive	26 to 35	NR	No	No	CRP	HCA and funisitis	63/100 (63)
Fisk et al. 1987 [29]	Saudi Arabia	Prospective cohort	55 (4)	Speculum examination—pooling of AF	26 to 36	NR	Selective, < 34 weeks	Selective, < 32 weeks	CRP	HCA	30/51 (59)
Danielian 1991 [30]	NR	Prospective cohort	17 (6)	NR	26–? (preterm)	NR	NR	NR	CRP	HCA	4/11 (36)
Yoon et al. 1996 [31]	South Korea	Prospective cohort	91 (28)	Pooling on speculum examination and nitrazine positive and fern test positive	20 to 37	NR	NR	NR	CRP	HCA and funisitis	35/63 (56)
Torbe 2007 [32]	?Poland	Prospective cohort	48 (0)	NR	24 to 34	Yes	Yes	None	CRP, PCT	HCA	14/48 (29)
Murtha et al. 2007 [33]	USA	Prospective cohort	122 (15)	Pooling of AF and nitrazine positive and ferning positive	22 to 34	Yes (all)	Selective (23 to 34 weeks)	NR	IL6	Funisitis	54/107 (50)
Smith et al. 2012 [34]	USA	Retrospective cohort	73 (0)	NR	20–37	Selective	Selective	NR	CRP	HCA	26/73 (36)
Perrone et al. 2012 [35]	Italy	Prospective cohort	66 (0)	Speculum exam; IGFBP-1 test	24 to 33	Yes	Yes	Yes	CRP	HCA and funisitis	24/66 (36)
Gulati et al. 2012 [36, 37]	India	Prospective cohort	45 (0)	NR	24 to 34	Yes	Yes	NR	IL6	HCA and funisitis	22/45 (49)
Canzoneri et al. 2012 [38]	USA	Prospective cohort	39 (0)	Pooling of AF and nitrazine positive and ferning positive	22 to 34	Yes (all)	Selective	No	IL6	funisitis	21/39 (54)
Oludag et al. 2014 [39]	Turkey	Prospective cohort	32 (0)	Speculum examination	24 to 34	Yes	Yes	NR	CRP, PCT	HCA	13/32 (41)
Aksakal et al. 2014 [40]	Turkey	Prospective cohort	50 (0)	Speculum examination or positive amnisure test	24 to 37	All	Selective, < 34 weeks	None	CRP	HCA and funisitis	24/50 (48)
Ronzino-Dubost et al. 2016 [41]	France	Prospective cohort	44 (14)	Speculum examination; IGFBP-1 test	24 to 34	Yes	Yes	Selective	CRP, PCT	HCA and funisitis	11/30 (37)
Thornburg et al. 2016 [42]	USA	Prospective	48	Speculum examination; fern and nitrazine test	23 to 33^+6^	Yes	Yes	Selective	PCT	HCA and funisitis	19/27 (70)
Kim et al. 2016 [43]	South Korea	Retrospective cohort	181 (35)	Pooling of AF on speculum examination	20 to 33^+6^	Yes	Yes	NR	CRP	HCA and funisitis	74/146 (51)
Stepan et al. 2016 [44]	Czech Republic	Prospective cohort	427 (41)	Speculum examination; IGFBP1 test when necessary	24 to 36^+6^	Yes	Yes	Yes	CRP	HCA and funisitis	238/386(62)
Kayem et al. 2017 [45]	France	Prospective cohort	184 (46)	History and speculum; other bedside test if necessary	< 37	Selective	NR	NR	CRP	HCA	85/138 (62)
Broumand et al. 2018 [46]/Seivani et al. 2017 [47]	Iran	Prospective cohort	48 (?)	Speculum examination; nitrazine and fern test	28 to 33	Yes	NR	NR	PCT	HCA	19/48 (40)
Martinez-Portilla 2019 et al. [48]	Mexico	Prospective cohort	64	Speculum examination, IGFBP-1 test	26 to 36^+6^	Yes	Yes	Yes	IL6	HCA	31/47 (66)
Asadi et al. 2019 [49]	Iran	Prospective, cohort	75 (23)	Pooling on speculum, fern and nitrazine tests	24 to 34	Yes	Selective	Selective	CRP, PCT	HCA	29/52 (56)
Park et al. 2019 [50]	South Korea	Retrospective cohort	82	Speculum; nitrazine test	23 to 34	Selective	Selective	Selective	CRP, IL6	HCA and funisitis	35/82 (43)
Totals											902/1717(37)

*GA* gestational age, *USA* United States of America, *NR* not reported, *HCA* histologic chorioamnionitis, *AF* amniotic fluid, *IGFBP-1* insulin-like growth factor binding protein-1

*Number given is the total number recruited, ‘excluded’ refers to participants whose index test or reference standard data was unavailable or not reported

*CRP* C-Reactive Protein, *PCT* Procalcitonin, *IL6* Interleukin 6

All studies reported data for preterm gestation (< 37 weeks) at the time of prelabour rupture of membranes (PROM), but the specific gestational age range for eligibility varied greatly among the included studies. Methods used to establish gestational age were unreported in most studies [26, 28–31, 34–36, 39, 41, 43, 45] except for 5 [27, 32, 40, 44, 49] which used a combination of last menstrual period and ultrasound. Where reported, diagnosis of PROM was made by clinical assessment (speculum examination) with some studies [27, 28, 31, 33, 35, 38, 40, 43–45, 49] conducting further confirmatory testing on all or some of the patients. Management of PPROM was largely expectant with monitoring of fetal well-being, surveillance for clinical features of chorioamnionitis and monitoring for signs of labour. Use of antibiotics, steroids and/or tocolytics where reported was universal or selective—dependent on gestational age or clinical features. Reasons for delivery included gestational age greater than 34 weeks [36, 40, 45], failed tocolysis or refractory labour [26–28, 35], completion of steroids or confirmed pulmonary maturity [26, 27, 44], foetal distress/abnormal cardiotocogram [26, 27, 35, 36, 44], suspected abruption [35] and/or other obstetric complications [36, 40, 44]. Six studies specified that clinical features of chorioamnionitis were an indication for delivery [26, 28, 29, 36, 44, 49]. According to the definitions of reference standard provided, 11 studies [26, 28, 31, 35, 36, 40–44, 50] reported the index test against a reference standard of HCA and/or funisitis, 10 [27, 29, 30, 32, 34, 39, 45, 46, 48, 49] reported HCA alone and 2 studies [33, 38] reported funisitis alone. Characteristics of included studies are outlined in Table 1. Studies evaluated the index tests over a wide range of cut-offs. More characteristics of index tests are provided in Additional file 4.

### Methodological quality of included studies

**Fig. 2:**
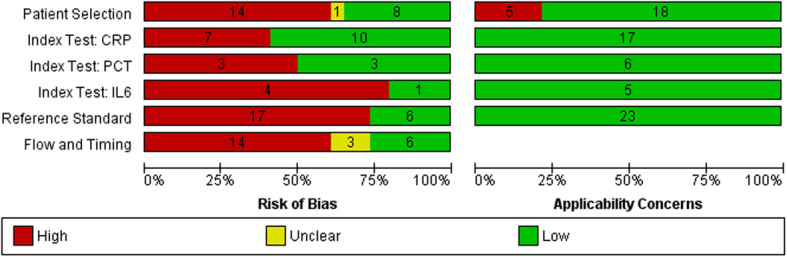
Risk of bias and applicability concerns graph [18] for included studies. CRP, C-reactive protein; PCT, procalcitonin; IL6, interleukin 6

Many studies were poorly reported, and 22 out of 23 were found to be at high risk of bias in at least 1 of the 4 domains of the QUADAS-2 (QUADAS-2 whiting) tool (Fig. 2, Additional file 5). In the ‘Patient selection’ domain, we judged 14 of the 23 studies to be at high risk of bias largely due to inappropriate exclusions such as excluding women based on duration after PPROM [35, 38], not explicitly excluding women with clinical features of chorioamnionitis at the time of PPROM or at the time of admission [26, 27, 31, 34, 38, 40], basing exclusions on availability or ability to perform other tests [31, 40, 45], excluding women due to missing data [34, 35, 50] and excluding women with common conditions and complications of pregnancy that often coexist with PPROM [32, 36, 39, 40]. In the ‘Index test’ domain, all tests were considered to be ‘blinded’ because maternal blood was collected before delivery and assessed on automated assays. Studies where the cut-offs used were not pre-specified [29, 31, 32, 35, 39, 43, 45, 49] but determined from the study data were also deemed to be at high risk of bias. Only 6 studies [27, 29, 33, 38, 40, 46] explicitly reported blinding in placental assessment. There were marked differences in the timing of collection of maternal blood, and many studies failed to report this clearly [26, 34, 36, 40]. We assumed *a* ≤ 72-h interval between maternal blood sampling and delivery to be appropriate as we felt the relationship between the index test and the outcome at placental assessment would be preserved. Only 11 studies [28–31, 33, 35, 36, 38, 41, 42, 49] had samples drawn within this interval. Studies that used samples obtained close to the time of admission or the time of PPROM would be at higher risk of bias due to variable lengths of latency after PPROM. All included studies had low concerns for applicability with regard to the index test and reference standard. In the ‘Patient selection’ domain, 5 studies [26, 27, 31, 35, 38] were judged to have high concerns for applicability as they did not explicitly report exclusion of contractions or advanced cervical dilatation (preterm labour).

### Findings

**Fig. 3:**
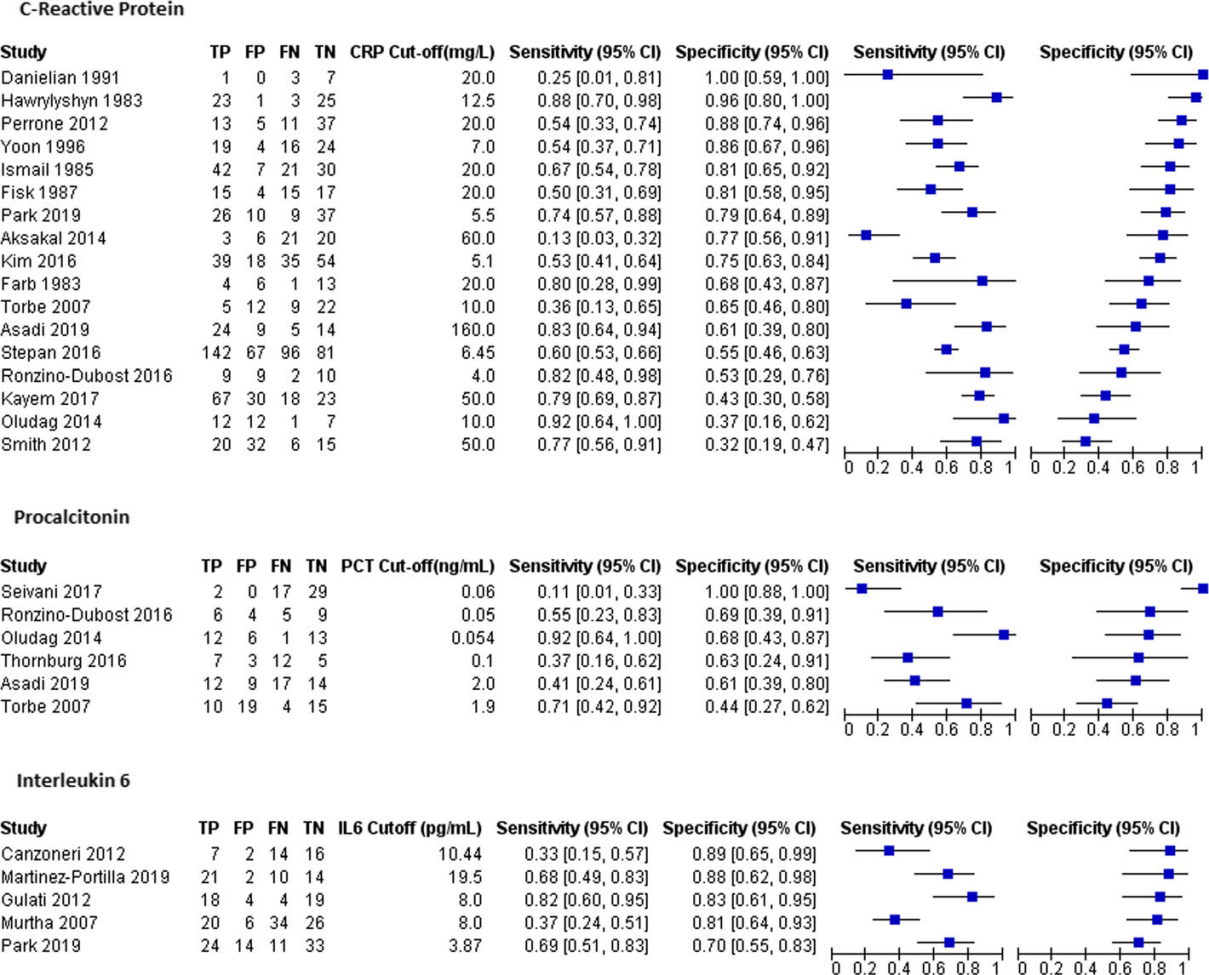
Forest plot showing sensitivity and specificity for included studies. TP—true positive, FP—false positive, FN—false negative, TN—true negative, CI—confidence interval, CRP—C-reactive protein, PCT—procalcitonin, IL6—interleukin 6. Studies are ordered by specificity in descending order for each index test group

**Fig. 4:**
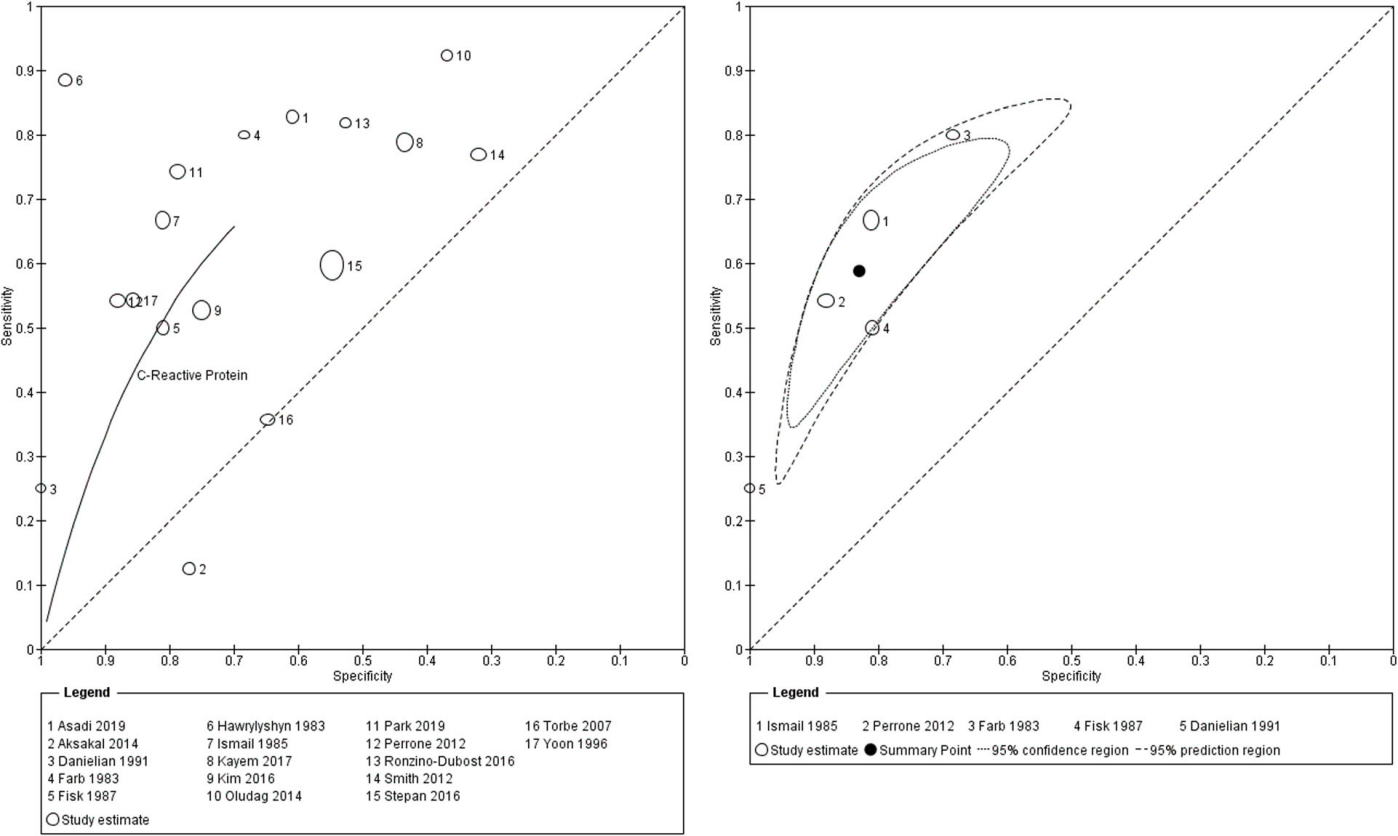
Summary ROC curve: C-reactive protein for histologic chorioamnionitis and/or funisitis; Curve 1 - C-reactive protein all studies. Curve 2 - C-reactive protein at 20 mg/L cutoff

**Fig. 5:**
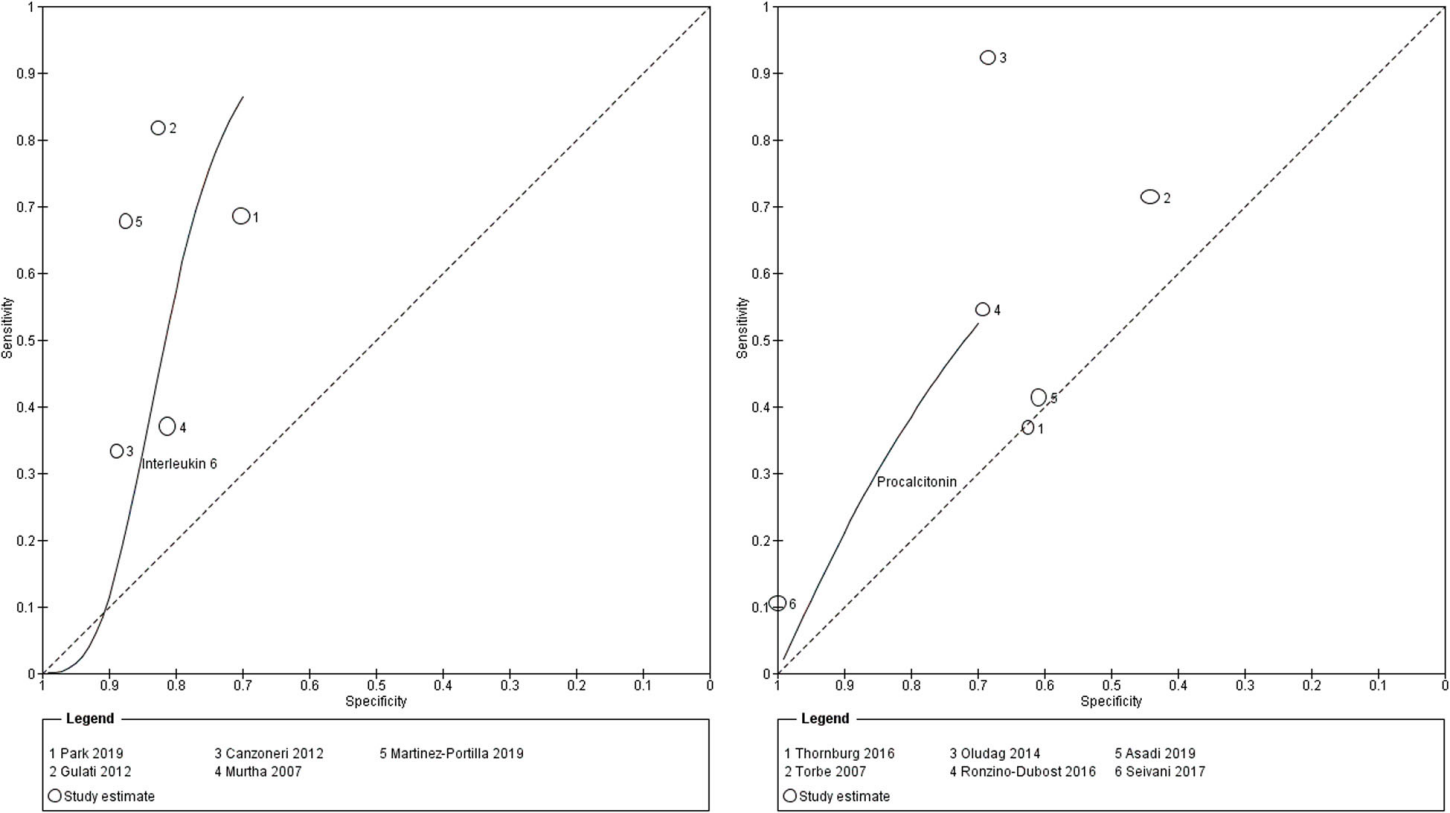
Summary ROC curves: interleukin 6 and procalcitonin for histologic chorioamnionitis and/or funisitis

Seventeen studies evaluated CRP as the index test, 6 evaluated the role of PCT and 5 evaluated IL6. Sensitivity and specificity pairs and their confidence intervals are demonstrated in Fig. 3. The forest plot shows wide variability in the sensitivity and specificity for each index test group. Studies reported data against a wide range of index test cut-offs (Fig. 3). Figures 4 and 5 show the various studies each plotted in ROC space as a single sensitivity-specificity point. The sizes of the individual points reflect the study sample size, and the scatter gives an impression of the heterogeneity in the findings. For CRP, 5 studies reported findings at a cut-off of 20 mg/L. A summary point of sensitivity and specificity is provided for this test group, and the large 95% prediction region reflects substantial heterogeneity. For the other test groups, a SROC curve is plotted for the range of sensitivity and specificity from the included studies. The closer the curve to the top left corner, the better the overall accuracy. The wide scatter of the study points in these plots suggests substantial heterogeneity.

### Findings of heterogeneity assessments

There was some heterogeneity as demonstrated by the 95% prediction region on the SROC (Fig. 4) for the studies reporting CRP at 20 mg/L. Further heterogeneity assessments revealed likely sources as interval between maternal blood sampling and delivery, nature of index test cut-off (predetermined or not), risk of bias score in the patient selection domain and assay type (Table 2, Additional file 6).

**Table 2:** Heterogeneity assessments and sensitivity analysis

	Heterogeneity assessments			Sensitivity analysis	
	Characteristic assessed	Findings	*p* value^ƚ^	Characteristic assessed	Findings
CRP (all cut-offs)	Predetermined cut-off	Studies using a predetermined cut-off had slightly lower accuracy	0.003	Gestational age range	Excluding studies that included GA < 24 weeks resulted in a slightly lower accuracy
	Interval between sampling and delivery	Studies > 72 h had lower accuracy	< 0.001	Applicability concerns in patient selection domain	Excluding studies with high concerns did not change the SROC curve
	Risk of bias in patient selection domain	Studies with low risk score had lower accuracy	< 0.001	Publication year after 2000	Excluding studies published before year 2000 yielded a slightly lower accuracy
	Assay type	Studies with CRP assays after standardisation (year 1993) had lower accuracy	< 0.001		
PCT	Predetermined cut-off	Studies using a predetermined cut-off had slightly lower accuracy	0.026	Gestational age range	Excluding studies that included GA < 24 had no effect
	Interval between sampling and delivery	No difference	0.178	Applicability concerns in patient selection domain	Excluding studies with high concerns resulted in a much lower accuracy and a change in shape of the curve
	Risk of bias in patient selection	Studies with low risk score had lower accuracy	< 0.001	Publication year after 2000	Not assessed as all studies were published after year 2000
IL6	Predetermined cut-off	Not assessed as 1 subgroup had < 2 studies		Gestational age range	Excluding studies that included GA < 24 weeks resulted in a slightly higher accuracy and change in shape of SROC curve
	Interval between sampling and delivery	Studies ≤ 72 h had lower accuracy	< 0.001	Applicability concerns in patient selection domain	Not assessed as all studies had low applicability concerns
	Risk of bias in patient selection	Not assessed as 1 subgroup had < 2 studies		Publication year after 2000	Not assessed as all studies were published after year 2000

More information is provided in Additional files 6 and 7

^ƚ^Likelihood ratio test

*CRP* C-Reactive Protein, *PCT* Procalcitonin, *SROC* Summary Receiver Operating Characteristic, *IL6* Interleukin 6

### Findings of sensitivity analysis

Sensitivity analysis for CRP were performed to assess the influence of including studies based on gestational age range, applicability concerns in the patient selection domain and year of publication. Year of publication was not assessed for PCT and IL6 as all studies were published after the year 2000. All IL6 studies had low applicability concerns in the patient selection domain, so this was not assessed. Results of the sensitivity analysis are given in Table 2 and Additional file 7.

Findings of this diagnostic review are summarised in the summary of findings table, Table 3.

**Table 3:** Summary of findings table

Maternal inflammatory markers for chorioamnionitis in preterm prelabour rupture of membranes(PPROM): a systematic review and meta-analysis of diagnostic test accuracy studies									
Question	In pregnant women with PPROM, can maternal serum inflammatory markers be used to diagnose chorioamnionitis?								
Population	Pregnant women with PPROM								
Studies	Any study design where the index test is compared against the reference standard								
Index test	C-reactive protein (CRP), procalcitonin (PCT) and interleukin 6 (IL6) assessed in maternal serum before delivery								
Reference standard	Histologic chorioamnionitis (HCA) and/ or funisitis								
Prevalence of disease	Median prevalence 50% (range 21–70%, IQR 38 to 57%) 23 studies with a total of 1717 pregnant women with PPROM, 902 of whom had HCA/funisitis								
Quality	Included studies were generally of poor quality with all studies at high risk of bias in at least one domain (QUADAS-2). There were few studies with high applicability concerns and only in the patient selection domain.								
Index test	Studies (participants)	Sensitivity (95% CI)	Specificity (95% CI)	Heterogeneity	Sensitivity analysis	Interpretation: assuming a patient population of 100 pregnant women with PPROM and prevalence of 50%*			
						Correctly diagnosed cases (TP)	Missed cases (FN)	Unnecessary interventions (FP)	True reassurance of no disease (TN)
CRP at 20 mg/L^†^	5 (252)	59% (47.7–69.0)	83% (74.0–89.2)	High heterogeneity despite common cut-off		30	21	9	42
CRP at all cut-offs^‡^	17 (1404)	59% (52.0–67.6)	80%	Partially explained by nature of cut-off used, sampling interval, risk of bias in the patient selection domain and type of CRP assay	Sensitive to gestational age range for study inclusion and year of publication	30	20	10	40
PCT at all cut-offs^‡^	6(231)	56% (49.9–68.9)	80%	Partially explained by nature of cut-off used and risk of bias in the patient selection domain of QUADAS-2	Sensitive to applicability concerns score in the patient selection domain of QUADAS-2	28	22	10	40
IL6 at all^‡^ cut-offs	5 (299)	52% (50.0–85.8)	80%	Partially explained by sampling interval	Sensitive to gestational age range for study	26	24	10	40

The results on this table should not be interpreted in isolation from the results in the main body of the text of the review

^*^Median prevalence from included studies

^†^Estimate from the summary point from bivariate analysis

‡Sensitivity derived from HSROC analysis assuming a specificity of 80% (false positive rate of 20%)

*CRP* C-Reactive Protein, *PCT* Procalcitonin, *IL6* Interleukin 6

## Discussion

### Main findings

The results of this review show the 3 tests have high false positive rates (low specificity) and high false negative rates (low sensitivity) in the diagnosis of histologic chorioamnionitis and/or funisitis (see Summary of findings table—interpretation). These findings are obtained in the background of few included studies with generally small sample sizes, poor quality assessments and substantial heterogeneity.

### Strengths and limitations

The findings of this review need to be evaluated with the knowledge of various strengths and weaknesses both of the included studies and those of the review methods. Included studies were few in number and generally had small sample sizes. This affects the precision and applicability of the findings, especially in the face of substantial heterogeneity. Studies were of poor quality with a high risk of bias in 1 or more domains. Poor reporting limited the assessment of methodological quality and applicability of many of the included studies. Findings of these studies are likely to be affected by various biases due to poor study design.

We have conducted this review following recommendations of the Cochrane group of diagnostic reviews [20] and following a prospectively registered protocol [14]. We employed a broad search strategy with search terms that did not include the reference standard and did not use a filter for ‘diagnostic studies’ [51]. However, a large proportion of potentially eligible studies were excluded due to inability to extract 2 × 2 data. Despite contacting authors of these studies, no additional data were obtained. We only included studies published in English and French and failed to obtain full texts of 6 articles. Our review was also limited to published studies only, limiting its representativeness.

Our review question limited the studies to those addressing a specific clinical condition in pregnancy, PPROM. This reduced chances of pooling together test accuracy indices that are different due to differences in patient characteristics and probability of disease [52]. All included studies had low concerns for applicability in the index test and reference standard domains. High applicability concerns arose in the patient selection domain particularly due to failure to explicitly exclude patients with preterm labour and perhaps due to poor reporting of inclusion criteria in some studies. We explored potential sources of heterogeneity where possible, but some subgroup analysis could not be carried out due to the few studies. We assumed the same shape (parallel curves) in comparing SROCs of subgroups due to the small number of studies—this would miss situations where the accuracy of the test varied with threshold in a different manner in the 2 subgroups compared.

Previous reviews [6, 7] examining the role of inflammatory markers in diagnosis of chorioamnionitis in PPROM had few studies, high between-study heterogeneity and differences in cut-offs that prevented pooled analysis. We identified more studies through our broader search criteria. These reviews [6, 7] also used methods of analysis that are no longer recommended. We used HSROC analysis [19, 20], a method that allowed pooling of studies with different cut-offs hence making efficient use of the data and maximising power [20]. We also assessed heterogeneity and identified likely sources. Despite these differences, our findings are in agreement with previous reviews that there is no evidence to support use of CRP, PCT or IL6 in the diagnosis of chorioamnionitis.

## Conclusions

### Implications for clinical practice

The proposed clinical role of the tests in PPROM is to guide interventions such as delivery or expectant management by appropriately identifying which pregnancies have chorioamnionitis. We have found insufficient evidence to recommend the use of either CRP, PCT or IL6 in maternal blood as a solitary test for the diagnosis of HCA/Funisitis in PPROM. Though it is relatively easy to obtain maternal blood for laboratory evaluation of these markers, the high false positive rates mean the tests should not be relied upon for important clinical decisions such as delivery. False positive results would have greater negative implications as they would result in iatrogenic preterm delivery with no indication. False positives at earlier gestations greatly could significantly impact neonatal outcome and survival.

Whether use of these tests should be recommended also depends on existence of and diagnostic performance of alternative tests in similar roles. Inflammatory markers in amniotic fluid may have better diagnostic performance than tests in maternal blood [53] but are limited by the complexity of amniotic fluid collection, increased costs and lower acceptability to women. Alternative approaches may be to combine these tests with other laboratory and clinical markers or to conduct serial tests [4]. This review did not examine these alternative tests and approaches.

### Implications for research

This review has demonstrated several weaknesses in the included studies and significant heterogeneity in findings that limit our ability to make reliable conclusions. There is need for better designed diagnostic accuracy studies where an effort is placed to reduce the various sources of bias as outlined in our quality assessments. In addition to assessing the role of the inflammatory marker, the contribution of other clinical and laboratory factors could be assessed jointly by regression modelling.

Several studies included in this report were poorly reported. Use of the standards for Reporting of Diagnostic Accuracy—STARD [54]—could reduce this and enable reviewers to correctly assess quality of studies and make more data available for review and meta-analysis.

## Supplementary information

**Additional file 1:.** Format: .docx Title “PRISMA-DTA Checklists” – Completed PRISMA-DTA checklist for the systematic review.

**Additional file 2:.** Format: .docx Title “Search strategy” – Table showing the search strategy for the review, Medline database on Ovid platform.

**Additional file 3:.** Format: .docx Title “Characteristics of Excluded Studies” – Table showing characteristics of studies excluded from the review due to missing or conflicting 2X2 data 1

**Additional file 4:.** Format: .docx Title “Characteristics of Index Tests in included studies” – Table showing the characteristics of all index tests in the included studies

**Additional file 5:.** Format: .png Title “Risk of Bias and Applicability Concerns Summary”

**Additional file 6:.** Format: .docx Title “Heterogeneity Assessments” – Figures and text showing and describing findings of the heterogeneity assessments

**Additional file 7:.** Format: .docx Title “Sensitivity Analysis” - Figures showing sensitivity analysis for studies evaluating C- reactive protein.

## Data Availability

The datasets used and analysed during the current study are available from the corresponding author on reasonable request.

## Abbreviations

CI: Confidence interval
CRP: C-reactive protein
FN: False negative
FP: False positive
HCA: Histological chorioamnionitis
HCA/Funisitis: Histologic chorioamnionitis and/or funisitis
HSROC: Hierarchical summary receiver operating characteristic
IL6: Interleukin 6
IQR: Inter-quartile range
NR: Not reported
PCT: Procalcitonin
PRISMA: Preferred Reporting Items for Systematic Reviews and Meta-Analyses
PRISMA-DTA: Preferred Reporting Items for Systematic Reviews and Meta-Analyses for Diagnostic Test Accuracy Studies
PPROM: Preterm prelabour rupture of membranes
PROM: Prelabour rupture of membranes
PROSPERO: International Prospective Register of Systematic Reviews
QUADAS-2: Quality Assessment of Diagnostic Accuracy Studies-2
ROC: Receiver operating characteristic
SROC: Summary receiver operating characteristic
STARD: Standards for Reporting Diagnostic Accuracy Studies
TN: True negative
TP: True positive
