# Supplementary material for: Maternal inflammatory markers for chorioamnionitis in preterm prelabour rupture of membranes: a systematic review and meta-analysis of diagnostic test accuracy studies
Source: Syst Rev. 2020 Jun 12;9:141. doi: 10.1186/s13643-020-01389-4 (PMC7293113; doi:10.1186/s13643-020-01389-4)
Supplement: Supplementary file 2 — Additional file 2:. Format: .docx Title “Search strategy” – Table showing the search strategy for the review, Medline database on Ovid platform. [file 13643_2020_1389_MOESM2_ESM.docx]

Additional file 2 - Search strategy

Database: Medline database, Ovid platform

Last search date 5^th^ January 2020

|  | Search |
| --- | --- |
| 1 | exp Fetal Membranes, Premature Rupture/ |
| 2 | rupture of membranes.af. |
| 3 | drainage of liquor.af. |
| 4 | amniorrhexis.af. |
| 5 | amniorrhea.af. |
| 6 | fetal membrane* .af. |
| 7 | foetal membrane* .af. |
| 8 | amniorrhoea.af. |
| 9 | amniorhexis.af. |
| 10 | amniotic sac.af. |
| 11 | amniotic fluid.af. |
| 12 | exp C-Reactive Protein/ |
| 13 | c reactive protein.af. |
| 14 | *crp.af. |
| 15 | procalcitonin.af. |
| 16 | pct.af. |
| 17 | exp Interleukin-6/ |
| 18 | interleukin 6.af. |
| 19 | il6.af. |
| 20 | il-6.af. |
| 21 | 1 or 2 or 3 or 4 or 5 or 6 or 7 or 8 or 9 or 10 or 11 |
| 22 | 12 or 13 or 14 or 15 or 16 or 17 or 18 or 19 or 20 |
| 23 | 21 and 22 |
| 24 | limit 23 to humans |
