# Supplementary material for: Maternal inflammatory markers for chorioamnionitis in preterm prelabour rupture of membranes: a systematic review and meta-analysis of diagnostic test accuracy studies
Source: Syst Rev. 2020 Jun 12;9:141. doi: 10.1186/s13643-020-01389-4 (PMC7293113; doi:10.1186/s13643-020-01389-4)
Supplement: Supplementary file 3 — Additional file 3:. Format: .docx Title “Characteristics of Excluded Studies” – Table showing characteristics of studies excluded from the review due to missing or conflicting 2X2 data 1 [file 13643_2020_1389_MOESM3_ESM.docx]

Additional file 3. Characteristics of Excluded Studies*

| Study | Country | Study Design | Gestational Age Range (weeks) | Index Test(s), and cut-off | Reference Standard / Outcome | Reason for Exclusion |
| --- | --- | --- | --- | --- | --- | --- |
| Evans 1980(1) | USA | Prospective cohort | ≤36 | CRP 2mg/dL | HCA | No 2x2 data for CRP vs HCA in PPROM subgroup (Study population includes term PROM, term and preterm labour, composite outcome of infectious morbidity) |
| Watts 1993(2) | USA | Prospective cohort | 22 -34 | CRP, 1.5mg/dL | HCA | No 2x2 data for PPROM subgroup  (Study population includes preterm labour) |
| Murtha 1996(3) | USA | Prospective cross-sectional | 22 -34 | IL6, 8pg/mL | HCA | No 2x2 data for IL6 vs HCA |
| Zou 2004(4) | China | Prospective cohort | 20 - 37 | CRP, 1.03mg/dL | HCA | No 2x2 data for PPROM subgroup  (Study population includes term PROM) |
| Skrablin 2007(5) | Croatia | Prospective cohort | 27-33 | CRP, 10.8mg/L  IL6, 27.5pg/mL | HCA | No 2x2 data for PPROM subgroup  (Study population includes preterm labour) |
| Yinon 2007(6) | Israel | Prospective cohort | 24 - 35 | CRP, cut-off not provided | HCA and funisitis | No 2x2 data for CRP vs HCA |
| Debieve 2011(7) | Belgium | Prospective Cohort | 24 -35 | CRP, 1mg/dL | HCA | No 2x2 data for CRP vs HCA |
| Oh 2011(8) | Korea | Retrospective cohort | 21 -35 (at birth) | CRP, 0.6mg/dL | HCA | No 2x2 data for subgroup with PPROM |
| Popowski 2011(9) | France | Prospective Cohort | >34 weeks | CRP, 5mg/L | HCA | No 2x2 data for PPROM subgroup (Study population includes term PROM) |
| Lee 2012(10) | Korea | Retrospective Cohort | <36 | CRP 8mg/L (4,8,12,20)) | HCA | No 2x2 data for the subgroup with PPROM |
| Wang 2012(11) | China | Prospective cohort |  | CRP, 4.4mg/L | HCA | No 2x2 data for PPROM subgroup  (Study population includes term PROM) |
| Cekmez 2013a(12) | Turkey | Prospective Cohort | 24-34 | CRP, 10.2pg/mL  IL6, 9.5pg/mL | HCA, ? Infectious morbidity | No 2x2 data for PPROM subgroup  (Unclear whether available data includes normal pregnant controls and whether it refers to HCA only or a composite outcome) |
| Cekmez 2013b(13) | Turkey | Prospective cohort | 24-34 | CRP 10.3pg/mL  IL6 9.6pg/mL | HCA, ?Infectious morbidity | Unclear whether 2x2 data refers to HCA only or a composite outcome |
| Jeon 2014(14) | Korea | Retrospective Cohort | Preterm, <37 weeks | CRP, 1.22mg/dL | HCA | No 2x2 data for the subgroup with PPROM |
| Kim 2014(15) | Korea | Retrospective Cohort | 24 -37 | CRP, 7.46mg/L | HCA and funisitis | No 2x2 data for the subgroup with PPROM |
| Park 2014(16) | Korea | Prospective Cohort | Preterm (at birth) | CRP, 0.7ng/mL | HCA | No 2x2 data for subgroup with PPROM |
| Xie 2015(17) | China | Retrospective cohort | <34 | CRP, 8mg/L | HCA | No 2x2 data CRP vs HCA or EONS |
| Kwak 2015(18) | Korea | Prospective Cohort | ≤ 37 | CRP, 0.8mg/dL | HCA | Unclear / Conflicting 2x2 data |
| Cho 2017(19) | Korea | Retrospective cohort | 20 – 36^+6^ | CRP 0.8mg/dL | HCA and Funisits | No 2X2 data for CRP vs HCA |
| Sayed 2016(20) | Egypt | Prospective cohort | 24-34 | IL6 8.5 pg/mL | HCA and Funisits | Conflicting 2X2 data |
| Deo 2016(21) | India | Prospective cohort | 20-37 | CRP 6mg/dL | HCA | No 2x2 data for the subgroup with PPROM |

** The table shows studies that were potentially eligible for inclusion but were not included because 2X2 data was unavailable or conflicting or unclear. Authors of these studies were contacted to provide data but no additional data was made available to the review.*

References

1. Evans MIH, S. N.; Devoe, L. D.; Angerman, N. S.; Moawad, A. H. C-reactive protein as a predictor of infectious morbidity with premature rupture of membranes. Am J Obstet Gynecol. 1980;138(6):648-52.

2. Watts DH, Krohn MA, Hillier SL, Wener MH, Kiviat NB, Eschenbach DA. Characteristics of women in preterm labor associated with elevated C-reactive protein levels. Obstetrics and Gynecology. 1993;82(4 I):509-14.

3. Murtha APG, P. C.; Jimmerson, C. E.; Roitman-Johnson, B.; Allen, J.; Herbert, W. N. Maternal serum interleukin-6 concentrations in patients with preterm premature rupture of membranes and evidence of infection. Am J Obstet Gynecol. 1996;175(4 Pt 1):966-9.

4. Zou H, Zhu J, Zhu JL, Zhang. The value of the soluable intercellular adhesion molecule-1 levels in matermal serum for determination of occult chorioamnionitis in premature rupture of membranes. J Huazhong Univ Sci Technolog Med Sci. 2004;24(2):154-7.

5. Skrablin H, Banovic V, Kralik S, Dijakovic A, Kalafatic DS, Lovric. Maternal plasma interleukin-6, interleukin-1beta and C-reactive protein as indicators of tocolysis failure and neonatal outcome after preterm delivery. J Matern Fetal Neonatal Med. 2007;20(4):335-41.

6. Yinon Y ZY, Weisz B, Mazaki-Tovi S, Sivan E, Schiff E, Achiron R. Fetal thymus size as a predictor of chorioamnionitis in women with preterm premature rupture of membranes. Ultrasound Obstet Gynecol. 2007;29(6):639-43.

7. Debieve F, Ska S, Williams O, Hutchings G, Bernard P, Grandjean P, et al. Evaluation of a universal real-time polymerase chain reaction for detection of amniotic fluid infection in premature rupture of membranes. Am J Perinatol. 2011;28(7):501-7.

8. Oh KH, Kim SN, Jeong EH, Lee SY, Yoon HYKJ, Park. Predictive value of intra-amniotic and serum markers for inflammatory lesions of preterm placenta. Placenta. 2011;32(10):732-6.

9. Popowski T, Goffinet F, Maillard F, Schmitz T, Leroy S, Kayem G. Maternal markers for detecting early-onset neonatal infection and chorioamnionitis in cases of premature rupture of membranes at or after 34 weeks of gestation: A two-center prospective study. BMC Pregnancy and Childbirth. 2011;11(26).

10. Lee KH, Jeong EH, Oh KJ, Ryu A, Park KUSY, Park. Relationship between maternal serum C-reactive protein, funisitis and early-onset neonatal sepsis. J Korean Med Sci. 2012;27(6):674-80.

11. Wang J, Zhang Y, Zhang H, Qu W, Lv J, Wang Y, et al. Increased sTREM-1 in pregnant women with premature rupture of membranes and subclinical chorioamnionitis. Mol Med Report. 2012;5(3):663-7.

12. Cekmez F, Ozkaya E, Pirgon O, Yilmaz Z, Yilmaz EA, Kaya G, et al. uPAR, IL-33, and ST2 values as a predictor of subclinical chorioamnionitis in preterm premature rupture of membranes. J Interferon Cytokine Res. 2013;33(12):778-82.

13. Cekmez F, Ozkaya E, Pirgon O, Yilmaz Z, Yilmaz EA, Korkmaz V, et al. Proadrenomedullin and serum amyloid A as a predictor of subclinical chorioamnionitis in preterm premature rupture of membranes. J Interferon Cytokine Res. 2013;33(11):694-9.

14. Jeon JHN, R.; Park, M. S.; Park, K. I.; Lee, C. Positive maternal C-reactive protein predicts neonatal sepsis. Yonsei Med J. 2014;55(1):113-7.

15. Kim MAL, Y. S.; Seo, K. Assessment of predictive markers for placental inflammatory response in preterm births. PLoS ONE. 2014;9(10):e107880.

16. Park CW, Kim SM, Park JS, Jun JK, Yoon BH. Fetal, amniotic and maternal inflammatory responses in early stage of ascending intrauterine infection, inflammation restricted to chorio-decidua, in preterm gestation. Journal of Maternal-Fetal and Neonatal Medicine. 2014;27(1):98-105.

17. Xie A, Zhang W, Chen M, Wang Y, Wang Y, Zhou Q, et al. Related factors and adverse neonatal outcomes in women with preterm premature rupture of membranes complicated by histologic chorioamnionitis. Medical science monitor : international medical journal of experimental and clinical research. 2015;21:390-5.

18. Kwak DW, Cho HY, Kwon JY, Park YW, Kim YH. Usefulness of maternal serum C-reactive protein with vaginal Ureaplasma urealyticum as a marker for prediction of imminent preterm delivery and chorioamnionitis in patients with preterm labor or preterm premature rupture of membranes. J Perinat Med. 2015;43(4):409-15.

19. Cho HY, Jung I, Kwon JY, Kim SJ, Park YW, Kim YH. The Delta Neutrophil Index as a predictive marker of histological chorioamnionitis in patients with preterm premature rupture of membranes: A retrospective study. PLoS ONE. 2017;12(3):e0173382.

20. Sayed Ahmed WA, Ahmed MR, Mohamed ML, Hamdy MA, Kamel Z, Elnahas KM. Maternal serum interleukin-6 in the management of patients with preterm premature rupture of membranes. The journal of maternal-fetal & neonatal medicine : the official journal of the European Association of Perinatal Medicine, the Federation of Asia and Oceania Perinatal Societies, the International Society of Perinatal Obstetricians. 2016;29(19):3162-6.

21. Deo DS, Jaiswar DSP, Sankhwar DPL, Kumari P, Singh DS. Evaluation of CRP as a Preindicative Marker in Women with Preterm Labour and Preterm Prelabour Rupture of Membrane (PPROM). International Journal of Life-Sciences Scientific Research. 2016;2(4).
