# Supplementary material for: Maternal inflammatory markers for chorioamnionitis in preterm prelabour rupture of membranes: a systematic review and meta-analysis of diagnostic test accuracy studies
Source: Syst Rev. 2020 Jun 12;9:141. doi: 10.1186/s13643-020-01389-4 (PMC7293113; doi:10.1186/s13643-020-01389-4)
Supplement: Supplementary file 4 — Additional file 4:. Format: .docx Title “Characteristics of Index Tests in included studies” – Table showing the characteristics of all index tests in the included studies [file 13643_2020_1389_MOESM4_ESM.docx]

Additional file 3. Characteristics of Index Tests in included studies.

| **Study Id** | **Test** | **Assay Type** | **Equipment and Manufacturer** | **Detection limit** | **Cut off** | **Predetermined cut off?** |
| --- | --- | --- | --- | --- | --- | --- |
| Farb 1983(1) | CRP | Nephelometric Immunochemistry | Beckman Instruments Inc., Fullerton, California | 1.8mg/L | 20mg/L | Yes |
| Hawrylyshyn 1983(2) | CRP | Rate nephelometry | Beckman Immunochemistry analyser, Beckman Instruments Inc., Fullerton, California | 6mg/L | 12.5mg/L | Yes |
| Ismail 1985(3) | CRP | Rate nephelometry | Beckman Immunochemistry analyser, Beckman Instruments Inc., Fullerton, California |  | 20mg/L | Yes |
| Fisk 1987(4) | CRP | Rate nephelometry | Beckman Instruments Inc., Fullerton, California | 6mg/L | 20, 30, 35, 40mg/L | No |
| Danielian 1991(5) | CRP | Rate nephelometry | Beckman Instruments Array Protein System |  | 20mg/L | Yes |
| Yoon 1996(6) | CRP | Antibody adsorption-particle agglutination assay(Seiken, Japan) | Hitachi 7470 Autoanalyzer, Hitachi, Japan | 1mg/L | 7mg/L | No |
| Torbe 2007(7) | CRP | Immuno-turbidimetry | Olympus AU 560, Olympus Diagnostica, Hamburg, Germany |  | 10mg/L | Yes |
| Perrone 2012(8) | CRP | Micro particle Enhanced Turbidimetric Immunoassay | Roche Diagnostic, Manheim, Germany | NR | 12, 20mg/L | No |
| Smith 2012(9) | CRP | NR | NR | NR | 50mg/L | Yes |
| Aksakal 2014(10) | CRP | NR | NR | NR | 60mg/L | Yes |
| Oludag 2014(11) | CRP | Immuno-turbidimetry | Abbott Diagnostics Architect c 16000 system, Abbott Diagnostics | NR | 10mg/L | Yes |
| Ronzino 2015(12) | CRP | NR | NR | NR | 4mg/dL | Yes |
| Kayem 2017(13) | CRP | NR | NR | NR | 5mg/dL | No |
| Stepan 2016(14) | CRP | High sensitivity Immunoturbidimetry | Roche, Basel, Switzerland | 0.3mg/L | 6.45mg/dL | No |
| Kim 2016(15) | CRP | Latex enhanced turbidimetric immunoassay | Denka, Seiken, Tokyo, Japan | NR | 5.1mg/dL | No |
| Torbe 2007(7) | PCT | Immunoluminometric assay | LUMI test, PCT kit, Brahms Diagnostica, Berlin Germany and Luminometer LIA-MAT system 300, BYK – Sangtec Diagnostic, Dietenbach, Germany | 0.1ng/mL | 1.9ng/mL | No |
| Oludag 2014(11) | PCT | Ultrasensitive immunoassay using TRACE(Time Resolved Amplified Cryptate Emission Technology) | Kryptor, Brahms | 0.019ng/mL | 0.054ng/mL | No |
| Broumand 2018(16) | PCT | Cobas e411 analyzer | Roche diagnostic kit | NR | 0.06ng/mL | Yes |
| Ronzino 2015(12) | PCT | Elisa immunological assay | KRYPTOR automated system compact, Brahms Company | NR | 0.05ng/mL | No |
| Murtha 2007(17) | IL6 | Ultrasensitive ELISA | Cytokine Core lab, Baltimore, Maryland | 1.2pg/mL | 1.8, 8pg/mL | No |
| Gulati 2012(18, 19) | IL6 | Standard ELISA, solid phase sandwich ELISA | Diaclone IL6 ELISA kit, Besancon, France | 2pg/mL | 8pg/mL | Yes |
| Canzoneri 2012(20) | IL6 | Ultrasensitive ELISA | Cytokine Core lab, Baltimore, Maryland | 1.2pg/mL | 1.98, 5.12, 10.44pg/mL | No |
| Martinez 2018(21) | IL6 | Immunoassay kit Th17 for humans | Thermo Scientific | NR | 19.5pg/mL | No |
| Park 2019(22) | IL6 | ELISA | R&D System, Minneapolis, Minnesota | 0.156pg/mL | 3.87pg/mL | No |

CRP, C reactive Protein; NR, Not Reported; PCT, Procalcitonin, IL6, Interleukin 6; ELISA, Enzyme Linked Immunosorbent Assay

**References**

1. Farb HF, Arnesen M, Geistler P, Knox GE. C-reactive protein with premature rupture of membranes and premature labor. Obstetrics and Gynecology. 1983;62(1):49-51.

2. Hawrylyshyn PB, P.; Milligan, J. E.; Soldin, S.; Pollard, A.; Papsin, F. R. Premature rupture of membranes: the role of C-reactive protein in the prediction of chorioamnionitis. Am J Obstet Gynecol. 1983;147(3):240-6.

3. Ismail MA, Zinaman MJ, Lowensohn RI, Moawad AH. The significance of C-reactive protein levels in women with premature rupture of membranes. American Journal of Obstetrics and Gynecology. 1985;151(4):541-4.

4. Fisk NM, Fysh J, Child AG, Gatenby PA, Jeffery H, Bradfield AH. Is C-reactive protein really useful in preterm premature rupture of the membranes? British Journal of Obstetrics and Gynaecology. 1987;94(12):1159-64.

5. Danielian PJ. CA 125 and preterm prelabour rupture of the membranes. Br J Obstet Gynaecol. 1991;98(8):835-6.

6. Yoon BH, Jun JK, Park KH, Syn HC, Gomez R, Romero R. Serum C-reactive protein, white blood cell count, and amniotic fluid white blood cell count in women with preterm premature rupture of membranes. Obstetrics and Gynecology. 1996;88(6):1034-40.

7. Torbe A. Maternal plasma procalcitonin concentrations in pregnancy complicated by preterm premature rupture of membranes. Mediators Inflamm. 2007;2007:35782.

8. Perrone G, Anceschi MM, Capri O, Galoppi P, Pizzulo S, Buccheri M, et al. Maternal C-reactive protein at hospital admission is a simple predictor of funisitis in preterm premature rupture of membranes. Gynecologic and Obstetric Investigation. 2012;74(2):95-9.

9. Smith EJ, Muller CL, Sartorius JA, White DR, Maslow AS. C-reactive protein as a predictor of chorioamnionitis. The Journal of the American Osteopathic Association. 2012;112(10):660-4.

10. Aksakal SE, Kandemir O, Altinbas S, Esin S, Muftuoglu KH. Fetal tyhmus size as a predictor of histological chorioamnionitis in preterm premature rupture of membranes. Journal of Maternal-Fetal and Neonatal Medicine. 2014;27(11):1118-22.

11. Oludag T, Gode F, Caglayan E, Saatli B, Okyay RE, Altunyurt S. Value of maternal procalcitonin levels for predicting subclinical intra-amniotic infection in preterm premature rupture of membranes. Journal of Obstetrics and Gynaecology Research. 2014;40(4):954-60.

12. Ronzino-Dubost V, Sananes N, Lavaux T, Youssef C, Gaudineau A, Lecointre L, et al. Evaluation of the interest of procalcitonin in the diagnosis of chorioamnionitis in preterm premature rupture of membranes. An observational and prospective study. Journal de Gynecologie Obstetrique et Biologie de la Reproduction. 2016;45(7):745-53.

13. Kayem G, Batteux F, Girard N, Schmitz T, Willaime M, Maillard F, et al. Predictive value of vaginal IL-6 and TNFalpha bedside tests repeated until delivery for the prediction of maternal-fetal infection in cases of premature rupture of membranes. European journal of obstetrics, gynecology, and reproductive biology. 2017;211:8-14.

14. Stepan M, Cobo T, Musilova I, Hornychova H, Jacobsson B, Kacerovsky M. Maternal Serum C-Reactive Protein in Women with Preterm Prelabor Rupture of Membranes. PLoS ONE. 2016;11(3):e0150217.

15. Kim SA, Park KH, Lee SM. Non-Invasive Prediction of Histologic Chorioamnionitis in Women with Preterm Premature Rupture of Membranes. Yonsei Med J. 2016;57(2):461-8.

16. Broumand F, Naji S, Seivani S. Predictive values of maternal serum levels of procalcitonin, ESR, CRP, and WBC in the diagnosis of chorioamnionitis in mothers with preterm premature rupture of membrane. Iranian Journal of Neonatology. 2018;9(2):50-60.

17. Murtha APS, T.; Hauser, E. R.; Swamy, G. K.; Herbert, W. N.; Heine, R. P. Maternal serum cytokines in preterm premature rupture of membranes. Obstet Gynecol. 2007;109(1):121-7.

18. Gulati S, Agrawal S, Raghunandan C, Bhattacharya J, Saili A, Agarwal S, et al. Maternal serum interleukin-6 and its association with clinicopathological infectious morbidity in preterm premature rupture of membranes: A prospective cohort study. Journal of Maternal-Fetal and Neonatal Medicine. 2012;25(8):1428-32.

19. Gulati S, Bhatnagar S, Raghunandan C, Bhattacharjee J. Interleukin-6 as a Predictor of Subclinical Chorioamnionitis in Preterm Premature Rupture of Membranes. Am J Reprod Immunol. 2012;67(3):235-40.

20. Canzoneri BJ, Grotegut CA, Swamy GK, Brancazio LR, Sinclair T, Heine PR, et al. Maternal serum interleukin-6 levels predict impending funisitis in preterm premature rupture of membranes after completion of antibiotics. Journal of Maternal-Fetal and Neonatal Medicine. 2012;25(8):1329-32.

21. Martinez-Portilla RJ, Hawkins-Villarreal A, Alvarez-Ponce P, Chinolla-Arellano ZL, Moreno-Espinosa AL, Sandoval-Mejia AL, et al. Maternal Serum Interleukin-6: A Non-Invasive Predictor of Histological Chorioamnionitis in Women with Preterm-Prelabor Rupture of Membranes. Fetal diagnosis and therapy. 2019;45(3):168-75.

22. Park JW, Park KH, Lee JE, Kim YM, Lee SJ, Cheon DH. Antibody Microarray Analysis of Plasma Proteins for the Prediction of Histologic Chorioamnionitis in Women With Preterm Premature Rupture of Membranes. Reprod Sci. 2019.

1. Farb HF, Arnesen M, Geistler P, Knox GE. C-reactive protein with premature rupture of membranes and premature labor. Obstetrics and Gynecology. 1983;62(1):49-51.

2. Hawrylyshyn PB, P.; Milligan, J. E.; Soldin, S.; Pollard, A.; Papsin, F. R. Premature rupture of membranes: the role of C-reactive protein in the prediction of chorioamnionitis. Am J Obstet Gynecol. 1983;147(3):240-6.

3. Ismail MA, Zinaman MJ, Lowensohn RI, Moawad AH. The significance of C-reactive protein levels in women with premature rupture of membranes. American Journal of Obstetrics and Gynecology. 1985;151(4):541-4.

4. Fisk NM, Fysh J, Child AG, Gatenby PA, Jeffery H, Bradfield AH. Is C-reactive protein really useful in preterm premature rupture of the membranes? British Journal of Obstetrics and Gynaecology. 1987;94(12):1159-64.

5. Danielian PJ. CA 125 and preterm prelabour rupture of the membranes. Br J Obstet Gynaecol. 1991;98(8):835-6.

6. Yoon BH, Jun JK, Park KH, Syn HC, Gomez R, Romero R. Serum C-reactive protein, white blood cell count, and amniotic fluid white blood cell count in women with preterm premature rupture of membranes. Obstetrics and Gynecology. 1996;88(6):1034-40.

7. Torbe A. Maternal plasma procalcitonin concentrations in pregnancy complicated by preterm premature rupture of membranes. Mediators Inflamm. 2007;2007:35782.

8. Perrone G, Anceschi MM, Capri O, Galoppi P, Pizzulo S, Buccheri M, et al. Maternal C-reactive protein at hospital admission is a simple predictor of funisitis in preterm premature rupture of membranes. Gynecologic and Obstetric Investigation. 2012;74(2):95-9.

9. Smith EJ, Muller CL, Sartorius JA, White DR, Maslow AS. C-reactive protein as a predictor of chorioamnionitis. The Journal of the American Osteopathic Association. 2012;112(10):660-4.

10. Aksakal SE, Kandemir O, Altinbas S, Esin S, Muftuoglu KH. Fetal tyhmus size as a predictor of histological chorioamnionitis in preterm premature rupture of membranes. Journal of Maternal-Fetal and Neonatal Medicine. 2014;27(11):1118-22.

11. Oludag T, Gode F, Caglayan E, Saatli B, Okyay RE, Altunyurt S. Value of maternal procalcitonin levels for predicting subclinical intra-amniotic infection in preterm premature rupture of membranes. Journal of Obstetrics and Gynaecology Research. 2014;40(4):954-60.

12. Ronzino-Dubost V, Sananes N, Lavaux T, Youssef C, Gaudineau A, Lecointre L, et al. Evaluation of the interest of procalcitonin in the diagnosis of chorioamnionitis in preterm premature rupture of membranes. An observational and prospective study. Journal de Gynecologie Obstetrique et Biologie de la Reproduction. 2016;45(7):745-53.

13. Kayem G, Batteux F, Girard N, Schmitz T, Willaime M, Maillard F, et al. Predictive value of vaginal IL-6 and TNFalpha bedside tests repeated until delivery for the prediction of maternal-fetal infection in cases of premature rupture of membranes. European journal of obstetrics, gynecology, and reproductive biology. 2017;211:8-14.

14. Stepan M, Cobo T, Musilova I, Hornychova H, Jacobsson B, Kacerovsky M. Maternal Serum C-Reactive Protein in Women with Preterm Prelabor Rupture of Membranes. PLoS ONE. 2016;11(3):e0150217.

15. Kim SA, Park KH, Lee SM. Non-Invasive Prediction of Histologic Chorioamnionitis in Women with Preterm Premature Rupture of Membranes. Yonsei Med J. 2016;57(2):461-8.

16. Broumand F, Naji S, Seivani S. Predictive values of maternal serum levels of procalcitonin, ESR, CRP, and WBC in the diagnosis of chorioamnionitis in mothers with preterm premature rupture of membrane. Iranian Journal of Neonatology. 2018;9(2):50-60.

17. Murtha APS, T.; Hauser, E. R.; Swamy, G. K.; Herbert, W. N.; Heine, R. P. Maternal serum cytokines in preterm premature rupture of membranes. Obstet Gynecol. 2007;109(1):121-7.

18. Gulati S, Agrawal S, Raghunandan C, Bhattacharya J, Saili A, Agarwal S, et al. Maternal serum interleukin-6 and its association with clinicopathological infectious morbidity in preterm premature rupture of membranes: A prospective cohort study. Journal of Maternal-Fetal and Neonatal Medicine. 2012;25(8):1428-32.

19. Gulati S, Bhatnagar S, Raghunandan C, Bhattacharjee J. Interleukin-6 as a Predictor of Subclinical Chorioamnionitis in Preterm Premature Rupture of Membranes. Am J Reprod Immunol. 2012;67(3):235-40.

20. Canzoneri BJ, Grotegut CA, Swamy GK, Brancazio LR, Sinclair T, Heine PR, et al. Maternal serum interleukin-6 levels predict impending funisitis in preterm premature rupture of membranes after completion of antibiotics. Journal of Maternal-Fetal and Neonatal Medicine. 2012;25(8):1329-32.

21. Martinez-Portilla RJ, Hawkins-Villarreal A, Alvarez-Ponce P, Chinolla-Arellano ZL, Moreno-Espinosa AL, Sandoval-Mejia AL, et al. Maternal Serum Interleukin-6: A Non-Invasive Predictor of Histological Chorioamnionitis in Women with Preterm-Prelabor Rupture of Membranes. Fetal diagnosis and therapy. 2019;45(3):168-75.

22. Park JW, Park KH, Lee JE, Kim YM, Lee SJ, Cheon DH. Antibody Microarray Analysis of Plasma Proteins for the Prediction of Histologic Chorioamnionitis in Women With Preterm Premature Rupture of Membranes. Reprod Sci. 2019.

References
