# Supplementary figures and images for: Maternal inflammatory markers for chorioamnionitis in preterm prelabour rupture of membranes: a systematic review and meta-analysis of diagnostic test accuracy studies
Source: Syst Rev. 2020 Jun 12;9:141. doi: 10.1186/s13643-020-01389-4 (PMC7293113; doi:10.1186/s13643-020-01389-4)

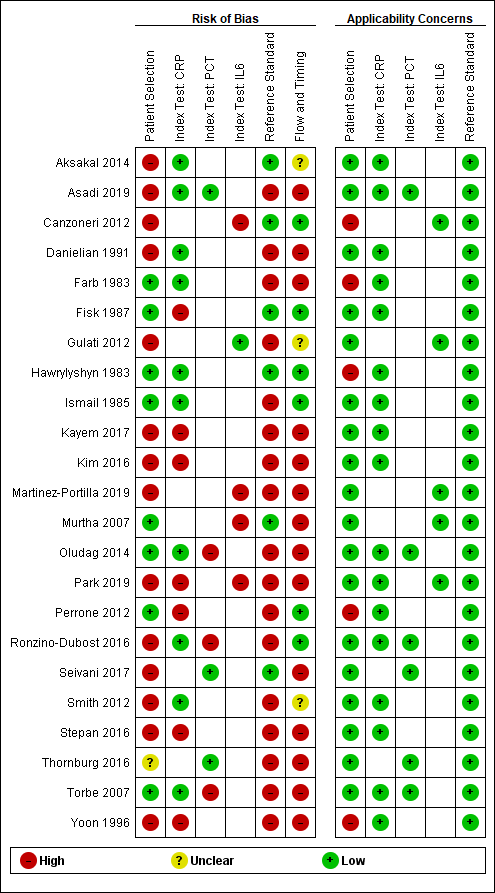

Supplement: Supplementary file 5 — Additional file 5:. Format: .png Title “Risk of Bias and Applicability Concerns Summary” [file 13643_2020_1389_MOESM5_ESM.png]
