# Supplementary material for: Maternal inflammatory markers for chorioamnionitis in preterm prelabour rupture of membranes: a systematic review and meta-analysis of diagnostic test accuracy studies
Source: Syst Rev. 2020 Jun 12;9:141. doi: 10.1186/s13643-020-01389-4 (PMC7293113; doi:10.1186/s13643-020-01389-4)

Additional file 5. Sensitivity Analyses

Sensitivity Analysis for Gestational Age in Studies Evaluating CRP in the Diagnosis of HCA/Funisitis.

All included studies


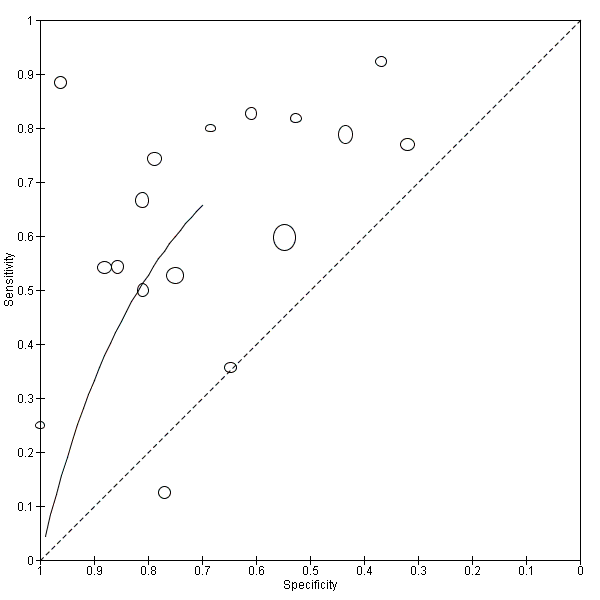


Studies without early (<24 weeks) gestations.


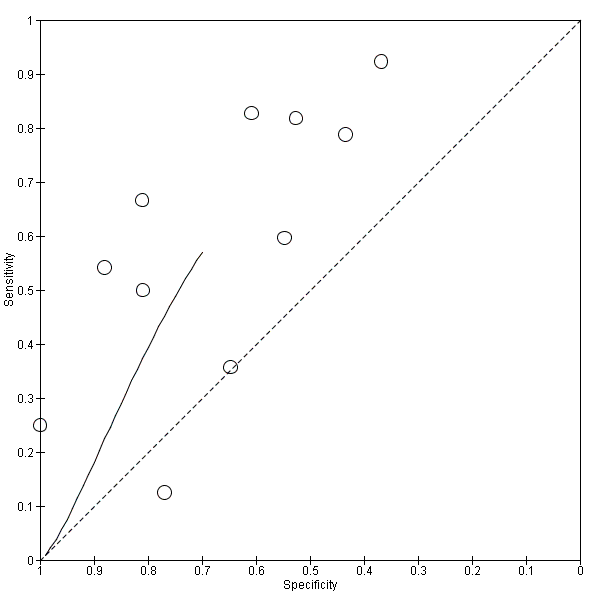


Sensitivity Analysis for Applicability Concerns in Patient Selection in Studies Evaluating CRP in the Diagnosis of HCA/Funisitis.

All included studies


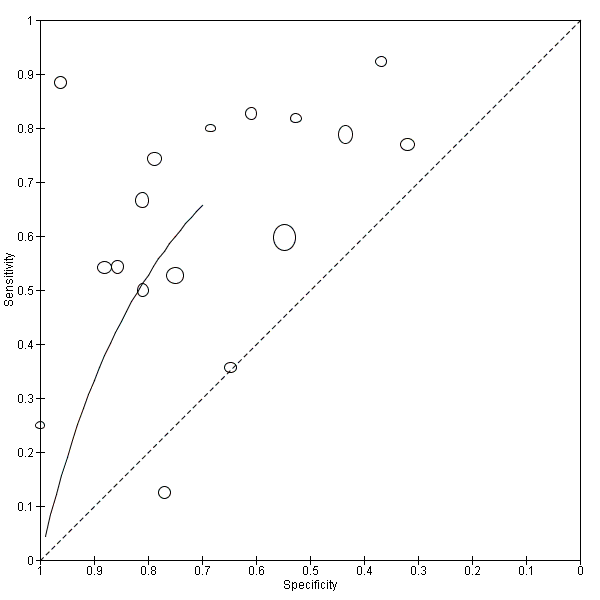


Studies with low applicability concerns in patient selection.


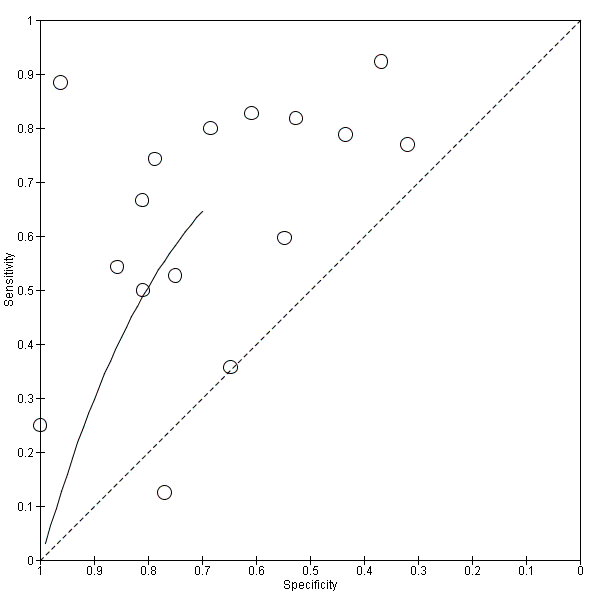


Sensitivity Analysis for Year of Publication in Studies Evaluating CRP in the Diagnosis of HCA/Funisitis.

All included studies


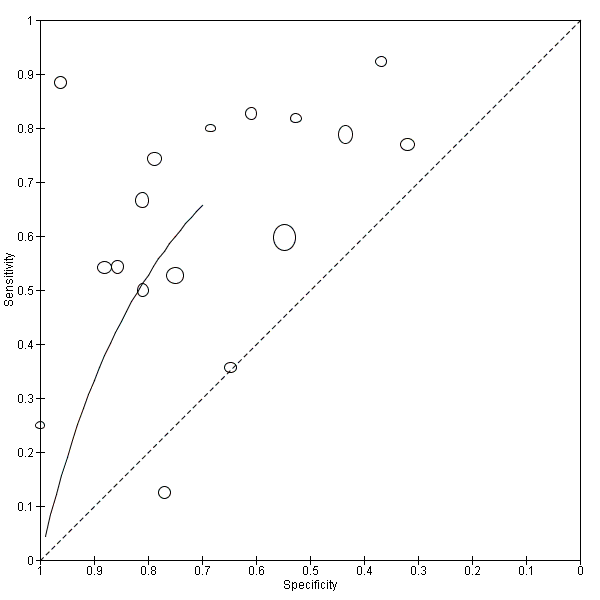


Studies published within 20 years of the review search date.
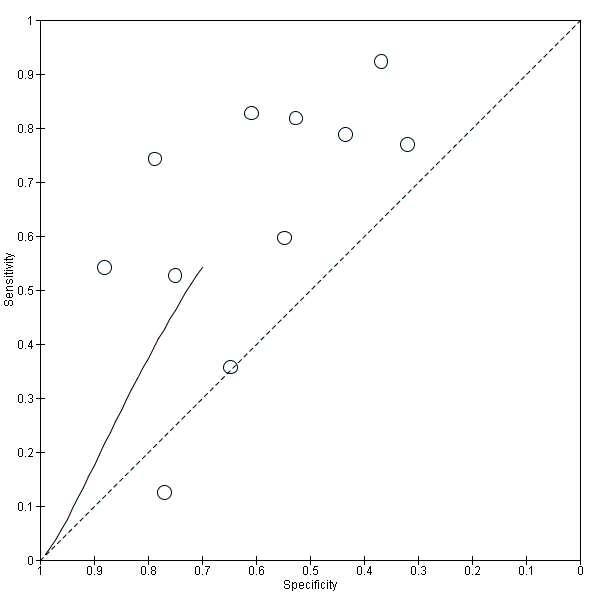


Sensitivity Analysis for Gestational Age in Studies Evaluating PCT in the Diagnosis of HCA/Funisitis.

All included studies


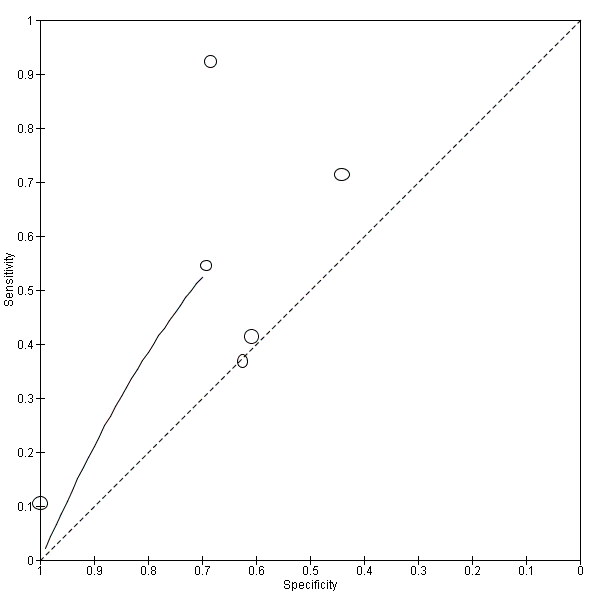


Studies without early (<24 weeks) gestations**.**


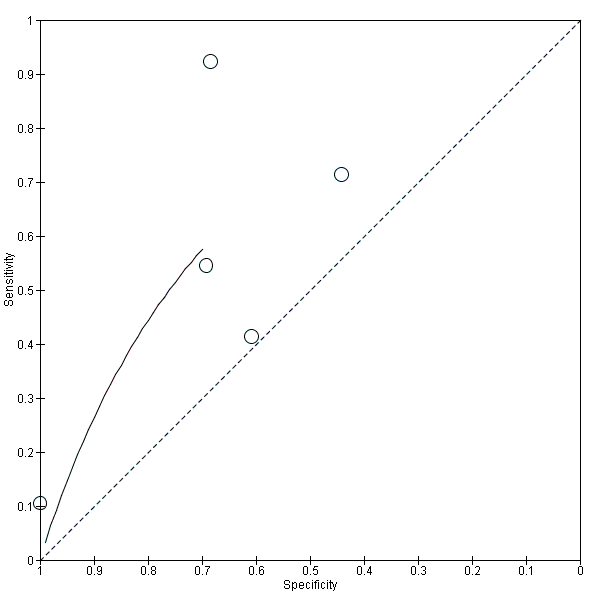


Sensitivity Analysis for Applicability Concerns in Patient Selection in Studies Evaluating PCT in the Diagnosis of HCA/Funisitis.

All Included Studies


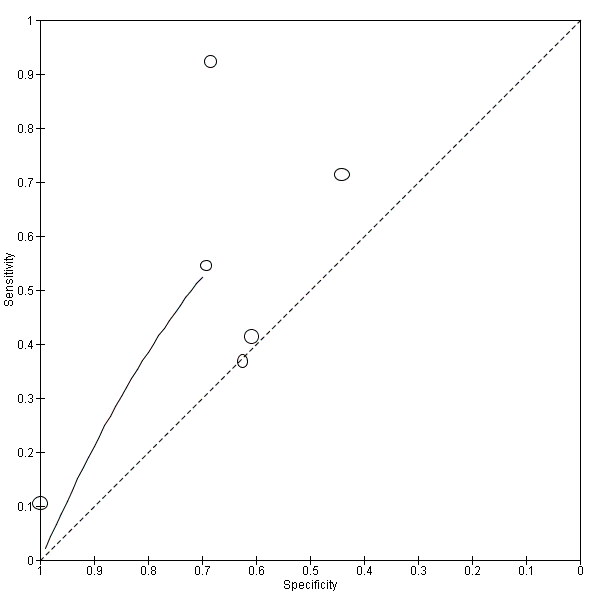


Studies with low applicability concernsin Patient selection


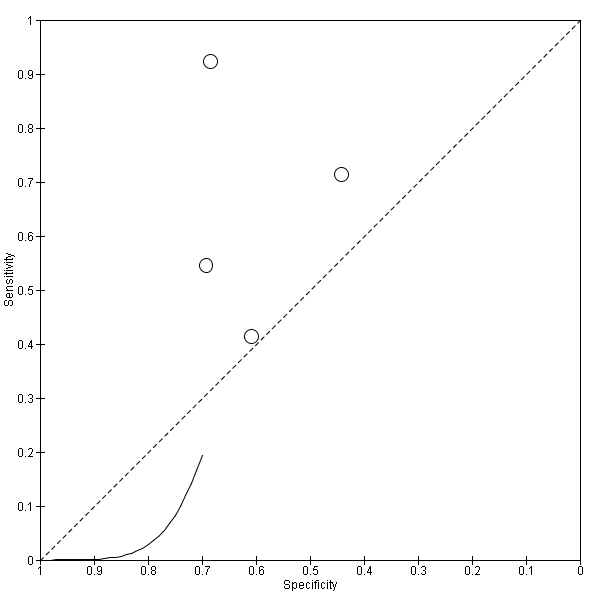


Sensitivity Analysis for Gestational Age in Studies Evaluating IL6 in the Diagnosis of HCA/Funisitis.

All Included Studies


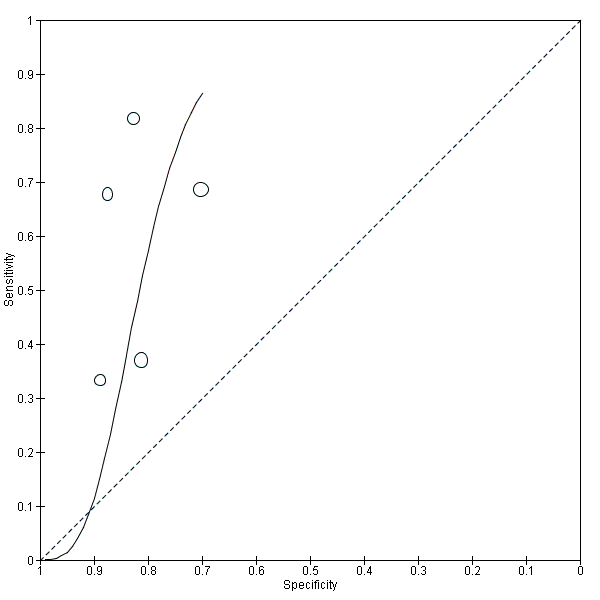


Studies without early (<24 weeks) gestations.


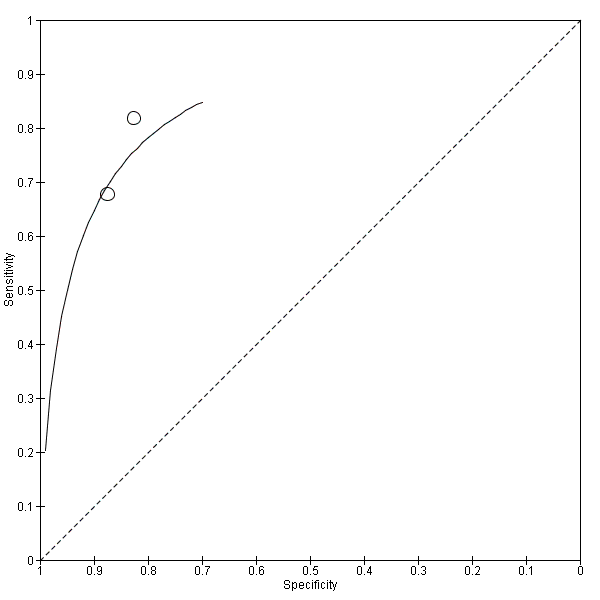

Supplement: Supplementary file 6 — Additional file 6:. Format: .docx Title “Heterogeneity Assessments” – Figures and text showing and describing findings of the heterogeneity assessments [file 13643_2020_1389_MOESM6_ESM.docx]
