# Supplementary material for: Maternal inflammatory markers for chorioamnionitis in preterm prelabour rupture of membranes: a systematic review and meta-analysis of diagnostic test accuracy studies
Source: Syst Rev. 2020 Jun 12;9:141. doi: 10.1186/s13643-020-01389-4 (PMC7293113; doi:10.1186/s13643-020-01389-4)
Supplement: Supplementary file 7 — Additional file 7:. Format: .docx Title “Sensitivity Analysis” - Figures showing sensitivity analysis for studies evaluating C- reactive protein. [file 13643_2020_1389_MOESM7_ESM.docx]

Additional file 4. Heterogeneity Assessments

1. Heterogeneity Assessments for Index Test C-Reactive Protein
   1. Assay type

Standardisation for CRP assays which was first performed in 1993^132^ was taken as the proxy for CRP assays before standardisation and after standardisation. The -2 log likelihoods of the 2 plots were compared with the ᵪ^2^ test yielding a p value of <0.001.

SROC Plots Comparing Studies Evaluating CRP in the Diagnosis of HCA and/or Funisitis, Subgroups: Assays Performed Before and After 1993.


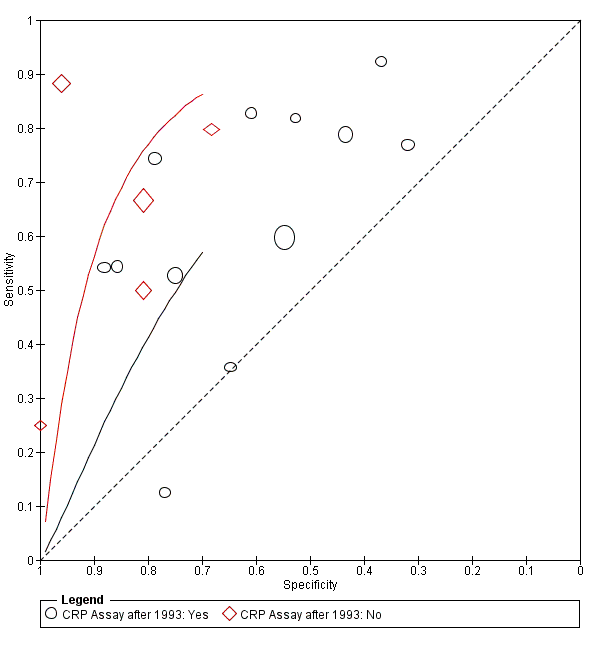


- 1. Pre-specified cut-off

Studies were grouped according to whether the cut-off used was pre-specified or whether it was determined from the study data. The -2 log likelihoods of the 2 plots were compared with the ᵪ^2^ test, p=0.003, indicating evidence for a difference in the two plots.

SROC Plots Comparing Studies Evaluating CRP in the Diagnosis of HCA and/or Funisitis, Subgroups: Pre-specified cut-off or not.


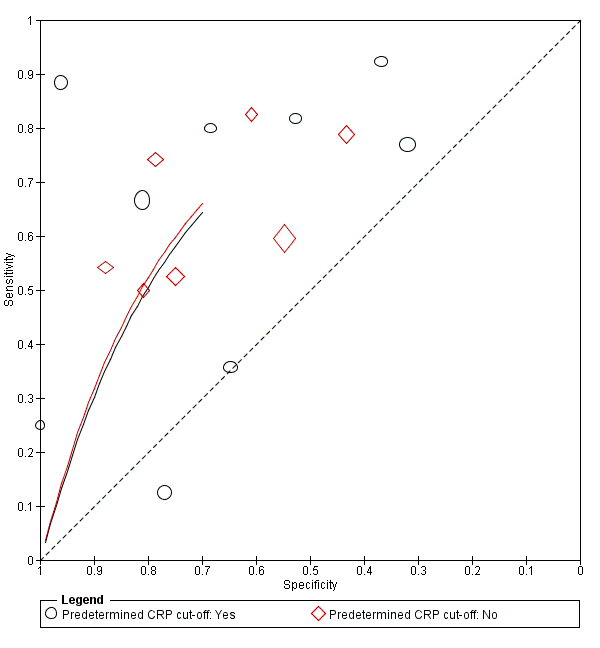


- 1. Interval from sampling to delivery

Studies were grouped according to sampling time with an interval of 72hours between sampling and delivery as the cut-off. The -2 log likelihoods of the 2 plots were compared with the ᵪ^2^ test. P<0.001 indicating that the 2 plots are different.

SROC Plots comparing Studies Evaluating CRP in the Diagnosis of HCA and/or Funisitis, Subgroups: Appropriate Sample Interval* or Not


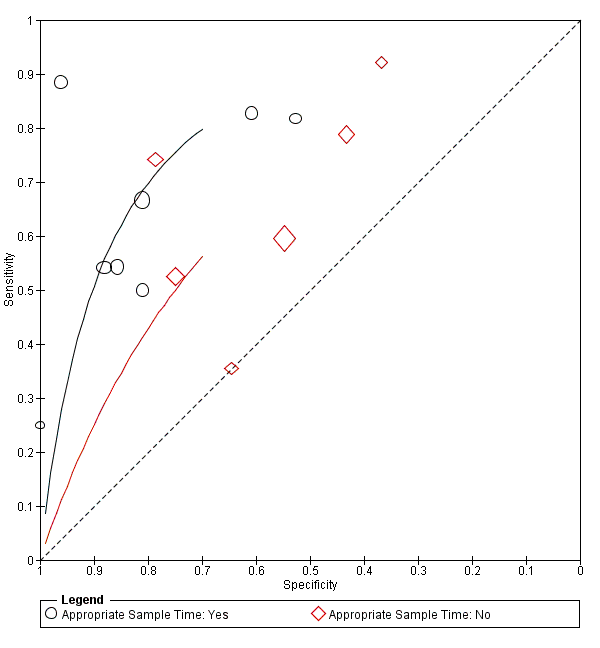


*Appropriate sample time - ≤72hours

- 1. Risk of Bias in Patient Selection

The judgements on risk of bias in the patient selection domain of the QUADAS-2^102^ tool were used to classify studies into 2 subgroups: high risk and low risk. The -2 log likelihoods of the 2 plots were compared with the ᵪ^2^ test. P<0.001 indicating evidence for a difference in the 2 plots.

SROC Plots comparing Studies Evaluating CRP in the Diagnosis of HCA and/or Funisitis, Subgroups: Low Risk and High Risk of Bias in the Patient Selection Domain


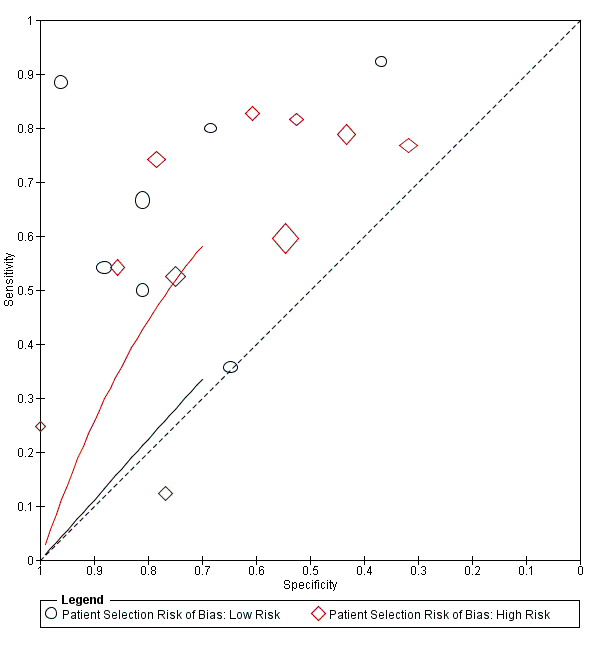


1. Heterogeneity Assessments for Index Test Procalcitonin
   1. Pre-specified cut-off

Studies were grouped according to whether the cut-off used was pre-specified or whether it was determined from the study data. The -2 log likelihoods of the 2 plots were compared with the ᵪ^2^ test, p=0.026, indicating evidence for a difference in the two plots.

SROC Plots Comparing Studies Evaluating PCT in the Diagnosis of HCA and/or Funisitis, Subgroups: Pre-specified cut-off or not.


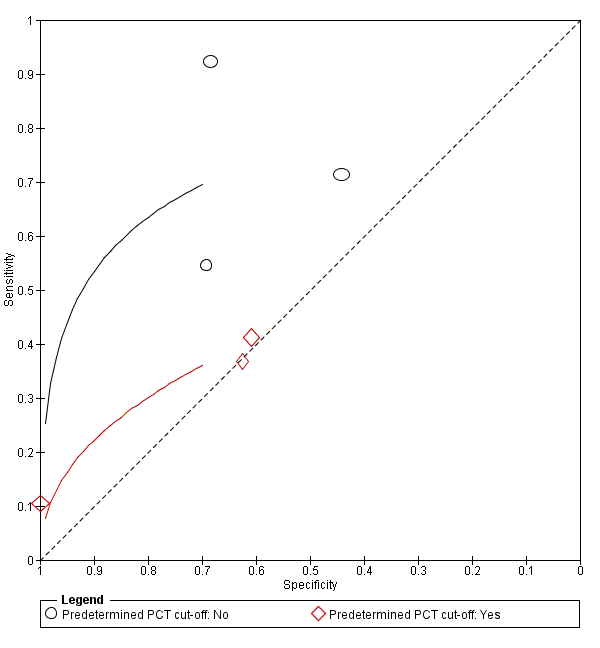


- 1. Interval from sampling to delivery

Studies were grouped according to sampling time with an interval of 72hours between sampling and delivery as the cut-off. The -2 log likelihoods of the 2 plots were compared with the ᵪ^2^ test. P=0.178 indicating no evidence that the 2 plots are different.

SROC Plots comparing Studies Evaluating PCT in the Diagnosis of HCA and/or Funisitis, Subgroups: Appropriate Sample Interval* or Not


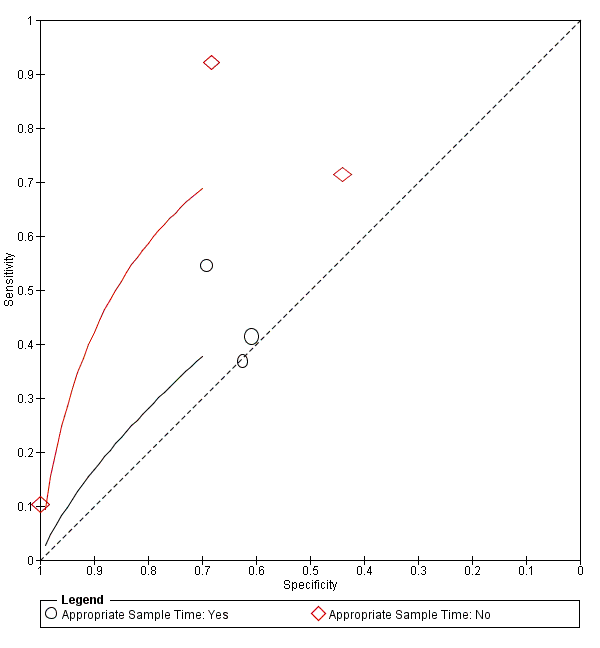


*Appropriate sample time - ≤72hours

- 1. Risk of Bias in Patient Selection

The judgements on risk of bias in the patient selection domain of the QUADAS-2^102^ tool were used to classify studies into 2 subgroups: high risk and low risk. The -2 log likelihoods of the 2 plots were compared with the ᵪ^2^ test. P<0.001 indicating evidence for a difference in the 2 plots.

SROC Plots comparing Studies Evaluating PCT in the Diagnosis of HCA and/or Funisitis, Subgroups: Low Risk and High Risk of Bias in the Patient Selection Domain


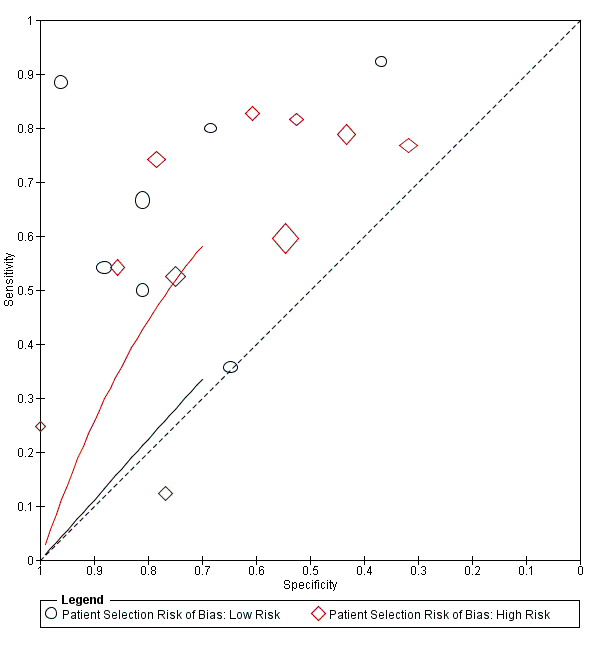


1. Heterogeneity Assessments for Index Test Interleukin 6
   1. Predetermined cut-off

This was not assessed as one of the subgroups (Predetermined cut-off =Yes) had only 1 study.

- 1. Interval from sampling to delivery

Studies were grouped according to sampling time with an interval of 72hours between sampling and delivery as the cut-off. The -2 log likelihoods of the 2 plots were compared with the ᵪ^2^ test. P<0.001 indicating evidence that the 2 plots are different.

SROC Plots comparing Studies Evaluating IL6 in the Diagnosis of HCA and/or Funisitis, Subgroups: Appropriate Sample Interval* or Not


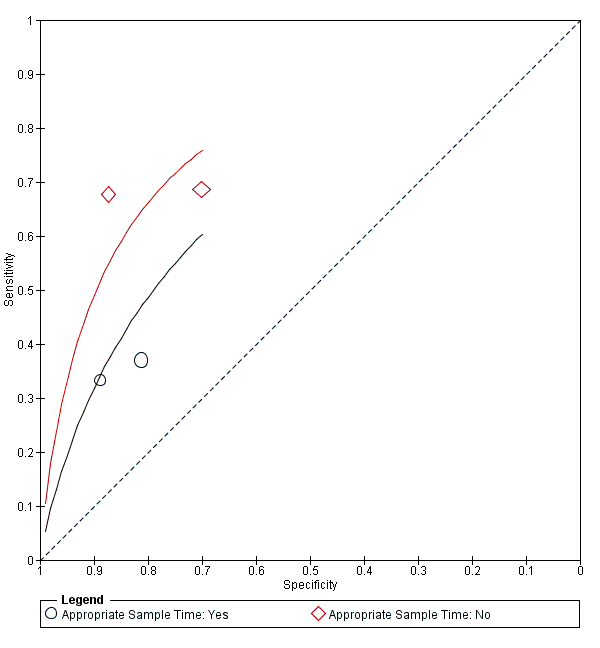


*Appropriate sample time - ≤72hours

- 1. Risk of Bias in Patient Selection

This was not assessed as one of the subgroups (Risk of bias =High) had only 1 study.
